# Windborne Dispersal of Arthropod Vectors as a Pathway for Lumpy Skin Disease Virus Introduction into South Korea: A Multi-Species, Source-Attributed Modelling Study

**DOI:** 10.3390/ani16182866

**Published:** 2026-09-11

**Authors:** Saleem Ahmad, Jinyoung Park, Jin-ho Jeong, Seung-Bum Kang, Kyung-Duk Min, Dae Sung Yoo

**Affiliations:** 1 Department of Veterinary Public Health, College of Veterinary Medicine, Chonnam National University, Gwangju 61186, Republic of Koreajhjeong9906@jnu.ac.kr (J.-h.J.); ksb3949bum@chonnam.ac.kr (S.-B.K.); 2 Laboratory of Veterinary Epidemiology, College of Veterinary Medicine, Chungbuk National University, Cheongju 28644, Republic of Korea; drdamus@cbnu.ac.kr (J.P.); kdmin@cbnu.ac.kr (K.-D.M.)

**Keywords:** lumpy skin disease, windborne vector dispersal, MaxEnt, Runge–Kutta trajectory model, South Korea, spatial permutation testing, space–time interaction

## Abstract

Lumpy skin disease (LSD) is a rapidly expanding transboundary vector-borne disease threatening livestock systems across Asia, with increasing risk of introduction into previously unaffected regions such as South Korea. This study developed an integrated, spatially explicit framework to assess the plausibility, timing and source-region structure of LSD virus (LSDV) introduction via windborne dispersal of infected arthropod vectors. Maximum Entropy models based on climatic, environmental, and topographic variables were used to determine habitat suitability for four major vector families (*Culex* spp., *Musca domestica*, *Stomoxys calcitrans*, and *Culicoides* spp.). All four were run via the transport simulation; *S. calcitrans* had the best experimental transmission efficiency, whereas *Culicoides* spp. provides the strongest evidence for passive long-distance transport. Livestock density and vector suitability were used to calculate the risk of LSD incidence in the adjacent source regions. A fourth-order Runge–Kutta trajectory model driven by ERA5 reanalysis winds at 850 hPa (about 1458 m) was used to predict windborne dissemination. This model included degradation of mechanically transmitted virus as well as survival limitations based on temperature, humidity, and wind. The simulated trajectories arrived in South Korea within the retention period of mechanically transported LSDV, with a median transit time of 18 h (interquartile range 10.0–30.3 h), 61.4% arriving within 24 h, and 87.1% within 48 h. North Korea accounted for 76.5% of weighted arrivals, with a median transit time of 11 h. Transit durations varied significantly by source location. For all four vector species, the predicted infectious arrival mass peaked in September and decreased thirteen-fold by November. Infected farms were found in regions of greater anticipated exposure than non-infected controls when compared to the October 2023 Korean epidemic (58.1% vs. 34.3% in the two highest risk groups; median exposure 0.214 versus 0.045; *p* < 0.001), although randomly relocating the dispersal surface reproduced comparable agreement in 23.6% of permutations, indicating that outbreak locations provide only limited validation of a pathway-specific introduction model. Random permutation of the dispersion surface produced a comparable result in 23.6% of permutations, indicating limited robustness of the observed spatial relationship to spatial randomization. With pairwise geographical and temporal distances significantly associated (Mantel r = +0.136, *p* = 0.013), spatiotemporal analysis revealed that the 74 reported outbreaks originated from a maximum of 25 introduction events over a 13-day period, consistent with significant farm-to-farm dissemination after introduction. The seasonal window, source-region structure, and transit timeframes of windborne LSDV introduction into South Korea are described in this paper. Reported epidemic sites only partially validate a pathway-specific introduction model since they reflect both the point of introduction and subsequent local transmission. This evaluation is pathway-specific and does not evaluate overall incursion risk; windborne transport is one of numerous possible introduction paths to consider.

## Simple Summary

Lumpy skin disease is a serious viral illness of cattle and water buffalo that causes skin nodules, fever, and significant losses in milk and meat production. Having spread from Africa across the Middle East, Europe, and Asia, it reached South Korea for the first time in October 2023. One route by which the virus can cross national borders is through infected insects—such as flies and midges—passively carried by wind currents over long distances. This study used computer models to test how plausible this pathway is for South Korea. We mapped where disease-carrying insects are most likely to thrive in the region, then simulated thousands of wind-driven insect journeys using detailed hourly weather data from 2023. The results showed that insects could reach South Korea in a median of 18 h—within the time the virus remains infectious—with North Korea accounting for 76.5% of projected arrivals. Risk was concentrated in late summer and early autumn. Farms affected in 2023 tended to lie in areas the model predicted to be at higher risk, although this association was not strong enough to confirm windborne introduction as the source of that outbreak. These findings can help animal health authorities decide when to intensify border surveillance and consider pre-emptive vaccination, potentially stopping an outbreak before it begins.

## 1. Introduction

Lumpy skin disease (LSD) is an emerging transboundary viral disease affecting cattle and water buffalo. It is caused by the LSD virus (LSDV), a member of the Capripoxvirus genus within the Poxviridae family [1]. The disease is characterized by high fever, generalized nodular skin lesions, lymphadenopathy, and peripheral edema, resulting in reduced milk yield, weight gain, reduced fertility, and reduced hide quality [2]. Although case fatality rates are low in endemic settings, LSD imposes substantial economic burdens through direct production losses, increased veterinary costs, prolonged trade restrictions, and mandatory international reporting obligations under the WOAH listing, which may lead to suspension of live animal and livestock product trade [3].

Originally described in Zambia in 1929 and confined to sub-Saharan Africa for decades, LSDV has undergone successive geographic expansions. The virus spread northward through Africa, became endemic in the Middle East during the early 2000s, and invaded the Balkans, the Caucasus, and Eastern Europe after 2012, extending into Russia and Central Asia [4]. Since 2019, LSD has spread across the Indian subcontinent and Southeast Asia, with outbreaks confirmed in India, Bangladesh, China, Vietnam, Thailand, and Indonesia [5,6,7]. Phylogeographic analyses suggest that the Asian epidemic was primarily driven by strains originating from the eastern Mediterranean and the Middle East through multiple independent transboundary transmission events. The confirmation of the first LSD outbreak in Japan in November 2024 [8] signaled an imminent and credible threat to the Korean Peninsula.

A key driver of LSDV spread is the interaction between climate change and arthropod vector biology. Rising temperatures and altered precipitation patterns are expanding the suitable habitat ranges of key LSDV-associated vectors, including *Culicoides* spp., Stomoxys calcitrans, and Aedes aegypti, toward higher latitudes and altitudes [9]. Species distribution modeling under future climate scenarios has projected substantial range expansions of *S. calcitrans* and Ae. aegypti across East Asia, including South Korea [10], reinforcing climate change as a structural, long-term driver of re-emerging vector-borne livestock diseases [9,11].

LSDV transmission is mediated predominantly by blood-sucking and synanthropic arthropods, including *S. calcitrans*, *Culicoides* spp., *Aedes* and *Culex* mosquitoes, and *Musca domestica* [12]. *Culicoides* spp. are particularly important for long-distance transboundary spread owing to their capacity for passive windborne transport over hundreds of kilometers, representing a credible insect-mediated introduction pathway during warm-season months [13]. Vector-laden livestock transport is an additional dispersal pathway within continental regions [14]. Collectively, wind-assisted arthropod dispersal and climate-mediated vector range expansion are growing pathways for LSDV introduction into disease-free countries.

Despite South Korea’s proximity to actively affected regions, particularly northeastern China and Japan, country-specific evidence for quantifying LSDV introduction risk is limited. Although ecological niche modeling studies have assessed LSD distribution at global and regional scales [7,15], country-specific analyses have been conducted only in Australia [15,16] and Thailand [17]. A comprehensive review of quantitative risk assessment frameworks further highlights the importance of integrating vector abundance, environmental suitability, and exposure metrics within a unified probabilistic approach [18]. However, no study has yet simultaneously integrated (i) habitat suitability modeling across the full range of LSDV-associated vectors; (ii) dynamic windborne atmospheric transport simulations using high-resolution meteorological reanalysis data; and (iii) livestock density-weighted exposure risk estimation for South Korea. Addressing this gap has become urgent given the eastward spread of the disease.

We focus on the windborne vector pathway because it is the only route whose spatial and temporal structure is determined by measurable physical processes and is therefore amenable to mechanistic simulation. Live animal movement, animal products, fomites, vector-laden transport and wildlife each contribute to overall incursion risk and are considered qualitatively in the Discussion (Section 4.5).

This study addressed the gap by developing an integrated, multi-component framework for assessing the plausibility and seasonal structure of LSDV introduction into South Korea. We (i) estimated habitat suitability for four key LSDV-associated vectors using Maximum Entropy (MaxEnt) ecological niche modeling; (ii) estimated spatial LSD occurrence risk across potential source regions by integrating vector suitability with susceptible host density; (iii) simulated windborne vector transport using a fourth-order Runge–Kutta (RK4) trajectory model driven by ERA5 meteorological reanalysis, weighted by vector survival and by decay of mechanically carried virus; and (iv) evaluated the ability of the resulting exposure surface to explain the location of the epidemic during 2023 in South Korea.

Specifically, we investigated three key questions: (i) whether windborne transport of infected arthropod vectors can reach South Korea within the estimated infectious retention period of mechanically contaminated vectors; (ii) which source regions contribute most to predicted arrivals and the associated transit times; and (iii) whether spatial variation in predicted windborne exposure is associated with the observed distribution of LSD outbreaks during the 2023 Korean epidemic.

## 2. Materials and Methods

### 2.1. Study Framework

**Figure 1:**
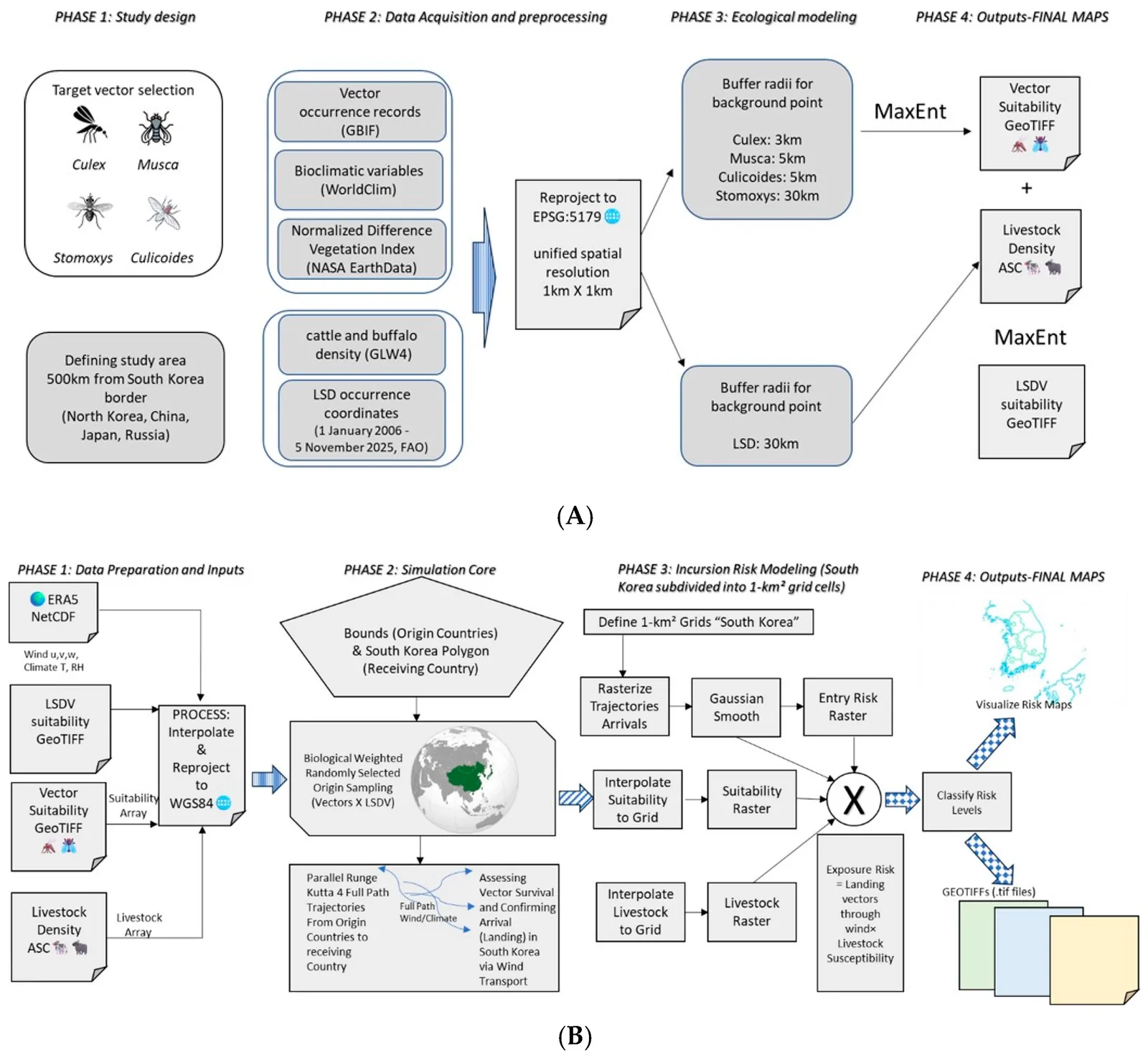
(**A**) Schematic diagram of the analytical workflow. Occurrence records, environmental variables, and host density data were preprocessed and used in a two-step MaxEnt modeling framework to determine vector suitability and generate a final LSDV habitat suitability map. (**B**) Integrated framework for modelling windborne incursion and exposure risk of LSDV into South Korea.

The overall analytical framework of the study is illustrated in Figure 1. For habitat suitability modeling (Figure 1A), occurrence records of four target vectors and LSD, climatic/environmental variables, and host density data were collected, preprocessed to a unified spatial resolution of 1 km, and used as inputs for MaxEnt modeling. Vector suitability outputs were subsequently combined with livestock density data in a second MaxEnt analysis to generate a final LSDV habitat suitability map. For windborne incursion modeling (Figure 1B), four sequential phases were implemented: (1) integration of environmental suitability, livestock density, and ERA5 meteorological data into a common spatial framework; (2) simulation of windborne vector transport using RK4 trajectory modeling with biologically weighted origin sampling and survival constraints; (3) conversion of survival-weighted landing events into gridded dispersal risk estimates integrated with environmental suitability and livestock density for exposure risk assessment; and (4) classification and visualization of final risk maps at a spatial resolution matched to that of the driving meteorological reanalysis.

### 2.2. Target Vectors and Study Area

Four key LSDV-associated vectors were selected based on their documented roles in transmission: *Culex* spp., *Musca domestica*, *Stomoxys calcitrans*, and *Culicoides* spp. [19]. Although *Culicoides* spp. can disperse up to 700 km via windborne transport [20], the maximum dispersal distance adopted for any LSDV-competent vectors is 500 km, based on entomological consultation [21]. Accordingly, the study area was defined as a 500 km buffer around the South Korean land border, computed as a metric buffer in EPSG:5179, encompassing North Korea and adjacent areas of China, Japan, and Russia.

All four vector species were carried through the transport simulation with species-specific thermal tolerance and virus-retention parameters. Evidence for the two components of the pathway is unevenly distributed across them: passive wind-assisted transport over hundreds of kilometers is best documented for *Culicoides* spp. [22,23], whereas experimental transmission efficiency is highest for *S. calcitrans* [24,25]. Results are presented for all four species, and this asymmetry is considered in Section 4.3.

### 2.3. Module A: Ecological Niche Modeling

#### 2.3.1. Data Acquisition

LSD occurrence records from 1 January 2006, to 5 November 2025, were obtained from the FAO EMPRES-i database [26], and vector occurrence data (2000–2025) were obtained from the Global Biodiversity Information Facility (GBIF) https://www.gbif.org/ (accessed on 15 December 2025). Cattle and buffalo density data were sourced from the Gridded Livestock of the World version 4 database [27]. Nineteen bioclimatic variables, solar radiation, wind speed, and elevation data were obtained from WorldClim (version 2.1) at a 30-arc-second resolution [28], and Normalized Difference Vegetation Index (NDVI) data were obtained from the MODIS MOD13A3 (version 6.1) product [29]. All variables are detailed in Table 1.

#### 2.3.2. Data Preprocessing

All spatial data were reprojected to EPSG:5179 and resampled to a 1 km^2^ resolution. Occurrence records outside the study area were excluded, yielding final datasets of 154 LSD outbreaks, 98 *Culex* spp., 530 *M. domestica*, 12 *S. calcitrans*, and 107 *Culicoides* spp. records. To address multicollinearity, bioclimatic variables with Pearson correlation coefficients > 0.7 or VIF > 5 were excluded, retaining Bio1, Bio3, Bio8, and Bio12. Solar radiation, wind speed, and NDVI were averaged across the study period, while elevation was treated as a static variable.

#### 2.3.3. Ecological Niche Modeling for Vector Habitat Suitability

MaxEnt models were developed for each vector using preprocessed occurrence records and selected predictor variables (Bio1, Bio3, Bio8, Bio12, solar radiation, wind speed, elevation, NDVI). Background point buffers were defined based on species-specific maximum active flight distances: 3 km for *Culex* spp. [30], 5 km for M. domestica [31] and *Culicoides* spp. [22], and 30 km for *S. calcitrans* [32]. Despite the small sample size, the 12 *S. calcitrans* records were retained because MaxEnt automatically restricts feature classes to prevent overfitting [33], and previous studies have demonstrated significant model performance with as few as 11 samples [34].

Because the resulting surfaces were each dominated by a single covariate (elevation for *S. calcitrans*, NDVI for *Culicoides* spp.; Section 3.1), they are interpreted as broad environmental gradients rather than as mechanistic habitat models.

#### 2.3.4. Spatial Risk Modeling of LSD in Source Regions

An additional MaxEnt model was developed to estimate the spatial risk of LSD occurrence in source regions. The model used LSD outbreak records as inputs and the four vector suitability maps together with cattle and buffalo density data as predictors. A 30 km background buffer radius was applied [35]. All analyses were conducted in R (version 4.4.1) [36] using the raster [37], dismo [38], sf [39], and dplyr [40] packages.

#### 2.3.5. Model Performance and Variable Contribution

Model predictive performance was evaluated using the area under the receiver operating characteristic curve (AUC) under restricted background conditions, reflecting species-specific dispersal distances, and random background conditions for comparison. Variable importance was assessed using percent contribution and permutation importance for all vector habitat suitability and LSD occurrence risk models.

### 2.4. Module B: Windborne Trajectory Simulation

#### 2.4.1. Meteorological Data and Preprocessing

Hourly meteorological data were obtained from the ERA5 reanalysis dataset [41], comprising zonal wind (u), meridional wind (v), vertical velocity (w), air temperature, and relative humidity, extracted from the pressure-level product at 850 hPa, corresponding to approximately 1458 m above mean sea level. This level lies within the nocturnal boundary layer in which windborne *Culicoides* transport is documented. Forward wind trajectories at this same 850 hPa level were used to quantify *Culicoides* dispersal during the 2006 north-western European bluetongue epidemic [22]. Because arthropod thermal tolerance thresholds are established for near-surface conditions, the sensitivity of results to the altitude at which survival is evaluated was assessed using a standard environmental lapse-rate correction (Section 2.6.1). Meteorological variables were accessed continuously through trilinear interpolation using the RegularGridInterpolator function, with time represented as sequential hourly ERA5 indices. The windborne trajectory simulation was performed in Python (version: 3.9.20). 

#### 2.4.2. Vector–LSDV Environmental Suitability Surfaces

Vector-specific combined suitability surfaces (vector habitat suitability × LSDV environmental suitability) were prepared as 1 km raster layers in EPSG:5179, reprojected to WGS84 (EPSG:4326) using bilinear resampling, and normalized to the range 0–1. Only grid cells with non-zero suitability values were retained for origin sampling and exposure estimation.

#### 2.4.3. Origin Sampling

Potential vector source origins were sampled from four regions: eastern China, Japan, the Russian Far East, and North Korea. For each region, 1200 origins were drawn with replacement from non-zero suitability grid cells within the 500 km buffer, with sampling probability proportional to suitability values. Because an equal number of origins was allocated to each region irrespective of its eligible area, origins are sampled more intensively per unit area in smaller regions; regional totals therefore reflect the sampling design rather than any assumption about relative source importance. Arrivals are reported by the country in which each origin falls rather than by sampling region (Section 3.6). Equal allocation across source regions is a stratified sampling design intended to provide comparable statistical power for each region; all inter-regional comparisons therefore use arrival rate (proportion of released trajectories resulting in arrival with viable virus) rather than arrival count.

#### 2.4.4. South Korea Land Mask

The South Korean land domain was defined using a strict administrative polygon derived from national boundary shapefiles (sig.shp), unified and reprojected to WGS84. All landing, dispersal, and exposure calculations were restricted to this land-only mask, excluding oceanic and offshore areas.

#### 2.4.5. Windborne Trajectory Modeling

Individual vector trajectories were simulated using an RK4 integration scheme with a 1 h time step and a maximum duration of 240 h (10 days). Horizontal displacement was driven by interpolated wind fields and converted from meters per second to degrees per hour as:

**dφ/dt = 3600·v/111,320;     dλ/dt = 3600·u/(111,320 · max(cos φ, 10^−6^))** (1)

Longitude values were wrapped to the interval [−180°, 180°]. Trajectories were initialized at sampled origins and integrated forward in time until termination. Simulated displacement was verified against the driving wind field: the ratio of implied to recorded wind speed had a median of 0.997 (interquartile range 0.921–1.084).

#### 2.4.6. Hazard-Based Survival Modeling

Vector survival was modeled multiplicatively using an hourly hazard framework incorporating thermal, humidity, and wind stress effects. Survival was set to a near-zero minimum (ε = 10^−6^) when temperature fell below 5 °C or exceeded 40 °C. Otherwise, a baseline hourly mortality hazard (m_0_ = 0.01) was modified by quadratic penalties for cold stress (<15 °C), heat (>30 °C), low humidity (<30%), moderate humidity (30–70%), high humidity (>90%), and excessive horizontal wind speed (>10 m s^−1^). Hourly survival probability was computed as:

**s = max(ε, exp(−m·Δt)), where m = m_0_ + Σ penalties** (2)

Cumulative survival was obtained as the product of hourly survival probabilities along each trajectory. All hazard and transport parameters are listed in Table 2.

#### 2.4.7. Virus Retention and Source Infection Prevalence

The trajectory model transports vectors; to represent virus introduction, arriving vectors were additionally weighted by the probability of still carrying infectious LSDV, modeled as w(t) = *p* · exp(−t/τ) for t ≤ t_max and zero thereafter.

The source term *p* was derived as the product of the probability that a vector had fed on an infected animal and the probability of acquiring virus given such a feed. Experimental estimates of acquisition are 0.23 from a clinically affected animal and 0.006 from a sub-clinically affected animal [42]. Assuming a clinical prevalence of approximately 1% in an affected source region gives *p* ≈ 2 × 10^−3^; a value of 1 × 10^−3^ was adopted. Because the model does not include a separate term for the probability of a vector being uplifted into a Windstream—estimated elsewhere at 10^−5^–10^−3^ per day [23]—*p* should be interpreted as the joint probability of infection and uplift, and its absolute magnitude treated as uncertain.

The retention constant τ distinguishes viral DNA persistence from retained infectivity. Infectious LSDV has been recovered from Stomoxys spp. immediately and 6 h after feeding, while viral DNA remained detectable to 48 h [43,44], and DNA has been reported on the proboscis for up to 8–9 days [24,45]. A 24 h interval between infectious and susceptible hosts was already too long to permit mechanical transmission [46], indicating that infectivity is lost before DNA becomes undetectable. We therefore adopted τ = 48 h as an upper bound on infectiousness and t_max = 96 h as a hard ceiling, and varied τ from 6 to 96 h in sensitivity analysis (Section 2.6.1). Because mechanical retention is short-lived, this term rather than the 240 h integration ceiling is the binding constraint on which arrivals contribute to predicted risk.

### 2.5. Module C: Risk Integration and Mapping

#### 2.5.1. Landing Definition and Post-Entry Dispersal

A trajectory was classified as having entered South Korea when it crossed the national land polygon. A landing event was defined as a trajectory that, at any land-intersecting time step, exhibited positive interpolated vertical velocity (w > 0), consistent with ERA5 downward-positive convention and representing forced descent or settling. For landed trajectories, all post-entry positions within South Korea were retained and weighted by cumulative survival at each time step and by retained infectiousness.

Each trajectory contributed at most one value per grid cell, taken as its maximum weight in that cell. In the submitted implementation, a trajectory contributed once per hour spent over a cell, so cells beneath slow-moving air accumulated weight for meteorological rather than epidemiological reasons.

#### 2.5.2. Dispersal Risk Mapping

South Korea was discretized into an approximately 1 km grid (0.009° resolution). Survival-weighted post-entry positions from all landed trajectories were aggregated into grid cells to generate a dispersal intensity surface. The resulting dispersal surface was normalized to the range [0, 1] and smoothed using a Gaussian filter (σ = 25 km), matched to the approximately 28 km native resolution of the driving reanalysis. Finer bandwidths exceed the resolution of the driving wind field (3). All values were masked to land areas only.

#### 2.5.3. Livestock Density

Cattle and buffalo density rasters were loaded from ASCII grids, normalized separately, summed, and re-normalized to the range [0, 1]. Livestock density was interpolated to the 1 km Korea grid using bilinear interpolation and restricted to land areas.

#### 2.5.4. Exposure Risk Estimation

Final livestock exposure risk was computed multiplicatively as:

**E = D_smooth × S_LSDV × H** (3)

where D_smooth is smoothed dispersal risk, S_LSDV denotes interpolated LSDV environmental suitability, and H denotes normalized livestock density. Exposure risk was normalized to the range 0–1 and masked to land. Because the dispersal component is limited by the resolution of the driving wind field while the suitability and host layers are available at 1 km, exposure risk is reported at the resolution of its limiting component.

#### 2.5.5. Risk Classification

Exposure and dispersal risk grids were classified into four ordinal categories using percentile thresholds derived from non-zero grid values: low (≤50th), moderate (50th–75th), high (75th–90th), and very high (>90th). This percentile-based classification avoided arbitrary absolute thresholds and ensured comparability across seasons and vectors.

### 2.6. Module D: Validation and Sensitivity Analysis

#### 2.6.1. Sensitivity Analysis

Nine survival parameters were varied individually across plausible ranges, together with offsets of 0 to +12 °C in temperature—spanning the environmental lapse-rate correction between the 850 hPa analysis level and near-surface conditions—±20 percentage points in relative humidity, and the virus retention constant from 6 to 96 h. Three outcomes were recorded: total survival- and retention-weighted arrival mass; the number of trajectories arriving with at least a 1% probability of surviving transit; and the Spearman rank correlation of the resulting dispersal surface against the baseline surface across all land cells. The last distinguishes parameters that rescale predicted risk from those that reorder its spatial pattern. Implementation details are given in Supplementary Materials.

#### 2.6.2. Validation Design

Predicted exposure was evaluated against farms reporting LSD during October 2023 at two spatial scales. At farm level, exposure values were extracted at case and control farm locations, with controls randomly sampled from the national cattle farm registry and matched to cases on livestock density decile. Nested logistic models were fitted with log livestock density, log habitat suitability and log dispersal intensity as sequential predictors, and the contribution of the dispersal surface assessed by likelihood-ratio test. At district level, outbreak counts per sigungu were modeled as a negative binomial process with the number of cattle farms as offset.

Because outbreak locations are spatially clustered through local transmission, conventional inference assuming independence is not valid. Statistical significance was therefore assessed by spatial permutation: the dispersal surface was randomly translated across the study area and each analysis repeated, generating an empirical null distribution that preserves the spatial autocorrelation structure of the predictor. Cases were additionally grouped into spatiotemporal clusters (15 km, 14 days) and the analysis was repeated using only the index case of each cluster. Residual spatial autocorrelation was assessed using Moran’s I. Analyses used SciPy [47], statsmodels [48] and scikit-learn [49]; the Mann–Whitney U test [50] and chi-square test [51] were applied where stated.

A Mantel test evaluating the association between paired geographical and temporal distances was used to evaluate the space–time interaction among reported outbreaks; significance was attained by permuting report dates among farms 9999 times. This separates local transmission from independent introductions into a common high-risk area while maintaining the marginal geographical and temporal distributions and testing solely their interaction. A Knox test was also used, but it yielded no results since all case pairings occurred within the 13-day reporting period, which was shorter than the incubation-based temporal window.

#### 2.6.3. Visualization and Outbreak Overlay

Trajectories were subsampled for visualization. Landed trajectories were rendered in color by source region, non-landing trajectories in light gray, and landing points in black. Confirmed LSDV outbreak locations were overlaid only on clean exposure risk maps to avoid visual clutter. All maps were exported at 600 dpi.

## 3. Results

### 3.1. Predicted Habitat Suitability of Vectors

**Figure 2:**
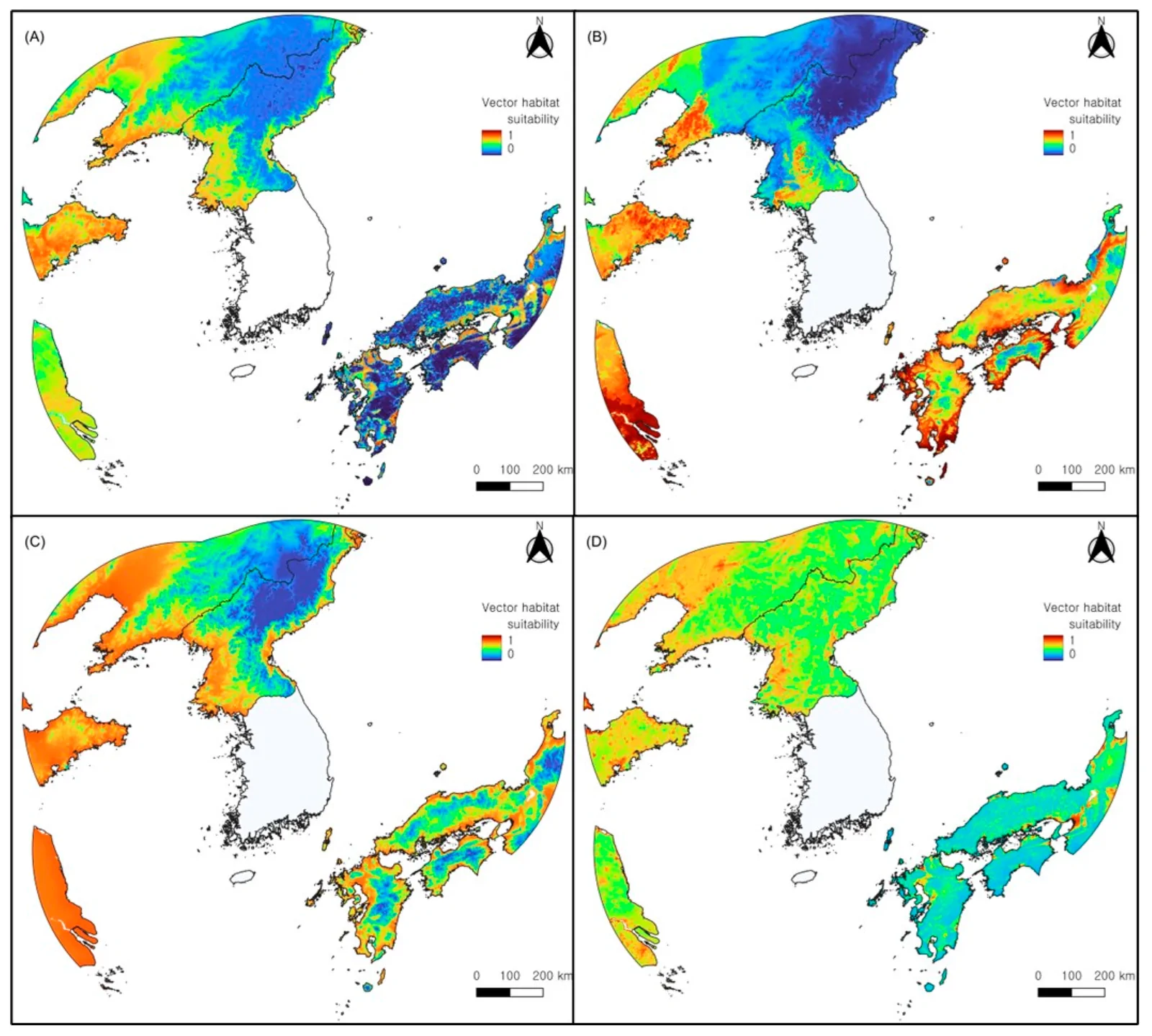
Predicted habitat suitability for four lumpy skin disease vectors within a 500 km radius of the South Korean border using MaxEnt modeling: (**A**) *Culex* spp., (**B**) *Musca domestica*, (**C**) *Stomoxys calcitrans*, (**D**) *Culicoides* spp. The color scale indicates the vector habitat suitability index from 0 (blue, low) to 1 (red, high).

Predicted habitat suitability differed markedly among the four vector species (Figure 2). *Culex* spp. exhibited high suitability along the western Korean coast and China’s Shandong Peninsula, with relatively low suitability in Japan. M. domestica exhibited a clear latitudinal gradient, approaching 0 in northern Korea and Primorsky Krai but nearing 1 in southern China and Japan. *S. calcitrans* demonstrated consistently high suitability across coastal regions, including the Korean Peninsula. *Culicoides* spp. showed the least spatial variation across the study area.

Key environmental drivers differed by species. NDVI and elevation were the primary determinants for *Culex* spp. For *M. domestica*, top contributors were wind speed (21.0%), Bio8 (18.6%), and solar radiation (17.9%). *S. calcitrans* distribution was almost exclusively driven by elevation (99.4%), while *Culicoides* spp. was dominated by NDVI (99.6%), with a minor contribution from annual precipitation (Bio12). Because each of these two surfaces is effectively determined by a single covariate, they represent broad environmental gradients rather than mechanistic habitat models, and are interpreted accordingly.

Because the *S. calcitrans* surface rests on 12 occurrence records and is almost entirely determined by elevation (99.4%), and the *Culicoides* spp. surface almost entirely by NDVI (99.6%), both should be regarded as preliminary. They are interpreted here as broad environmental gradients rather than as mechanistic habitat models.

### 3.2. Predicted Risk of LSD at Origin

**Figure 3:**
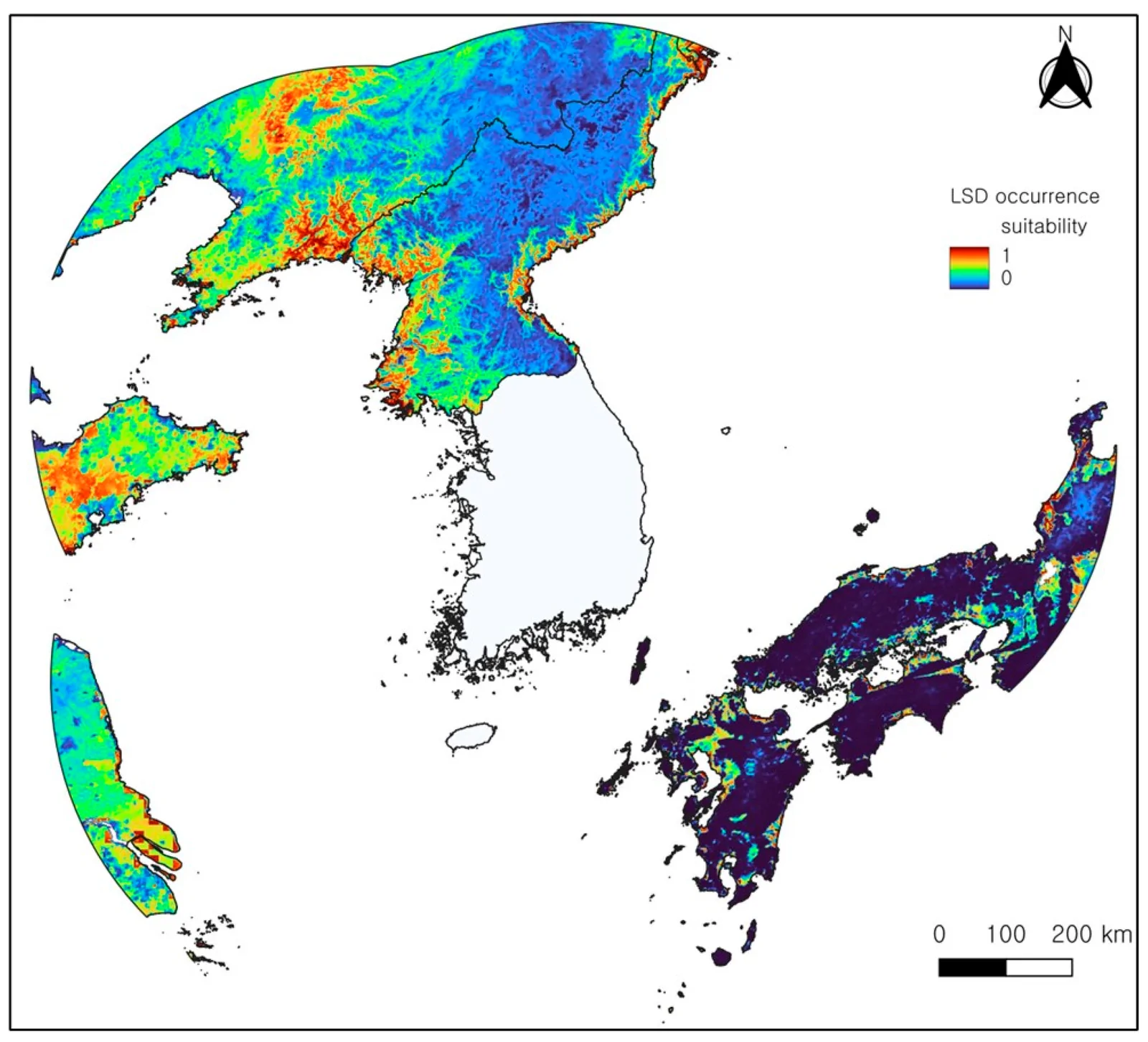
Predicted spatial suitability for lumpy skin disease occurrence. The color scale indicates the suitability index from 0 (blue, low) to 1 (red, high).

The LSD suitability map revealed substantial spatial heterogeneity across the study area (Figure 3). Suitability was highest along the western coast of North Korea, the Liaodong Peninsula, China’s Shandong Peninsula, and the southern coast of Primorsky Krai, Russia. Japan demonstrated generally low suitability, although localized high-risk hotspots were identified along its western and southern coastlines.

In the LSD risk model, *Culicoides* spp. habitat suitability was the dominant predictor (36.8%), followed by *Culex* spp. (29.2%), S. calcitrans (17.7%), and cattle density (15.3%). M. domestica contributed minimally (1.0%), and buffalo density had no influence (0%).

### 3.3. Model Performance

AUC values for individual vector models ranged from 0.609 to 0.705 under restricted background conditions. *Culicoides* spp. achieved the highest performance (0.705), followed by *Culex* spp. (0.658), *S. calcitrans* (0.650), and *M. domestica* (0.609). The integrated LSD occurrence risk model achieved an AUC of 0.827, substantially outperforming individual vector models. Under random background conditions, all models exceeded 0.9 (range: 0.905–0.974). The difference between restricted and random background values reflects the well-recognized inflation of AUC when background points are drawn from the full study extent; the restricted-background values are the appropriate basis for interpretation, and indicate modest discriminatory ability for the individual vector models.

### 3.4. Windborne Vector Transport and Survival Outcomes

**Figure 4:**
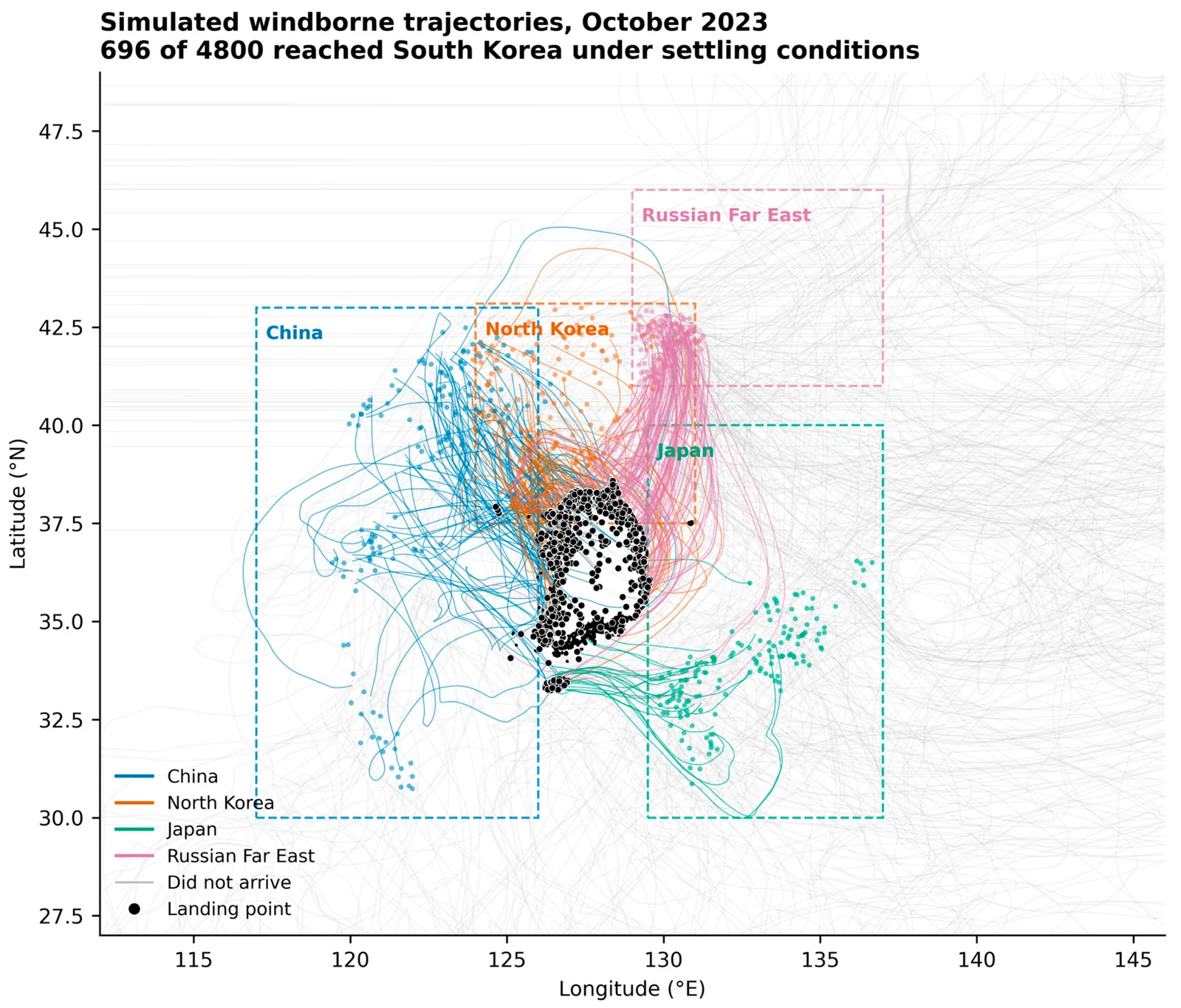
Simulated windborne trajectories, October 2023. Colored lines show paths of trajectories reaching South Korea under settling conditions, plotted from release to first landing; gray lines show a random subsample of trajectories that did not arrive. Black points mark landing locations. Dashed rectangles indicate source sampling domains.

**Figure 5:**
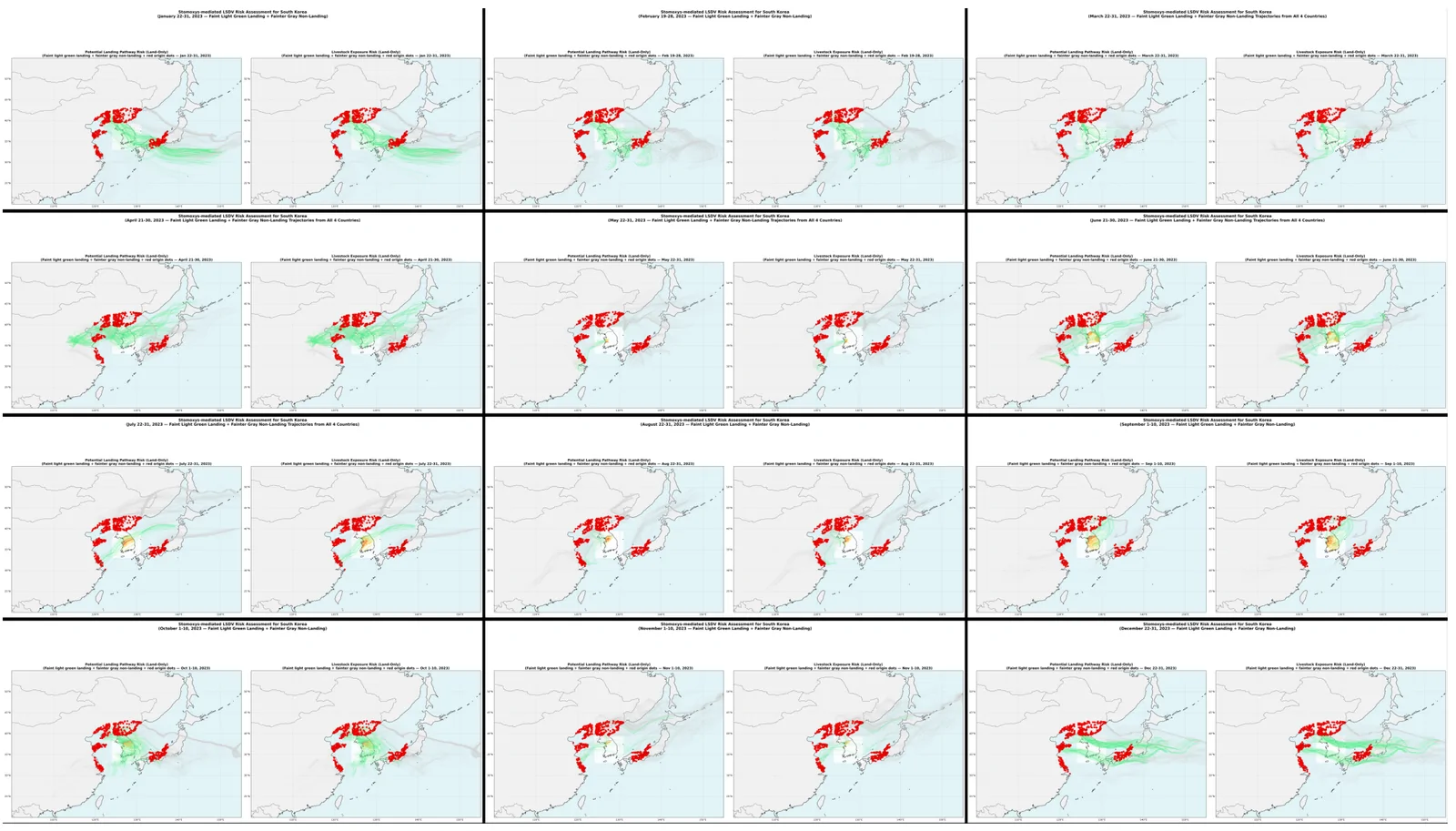
Monthly Stomoxys calcitrans-mediated LSDV transmission risk maps for South Korea, 2023. For each month the left panel shows potential landing pathways from 4800 simulated trajectories, and the right panel the corresponding livestock exposure risk distribution. Red dots are the origin points in the origin countries; Light green lines denote landing trajectories that reached South Korea; light grey lines denote trajectories that passed by without landing.

**Figure 6:**
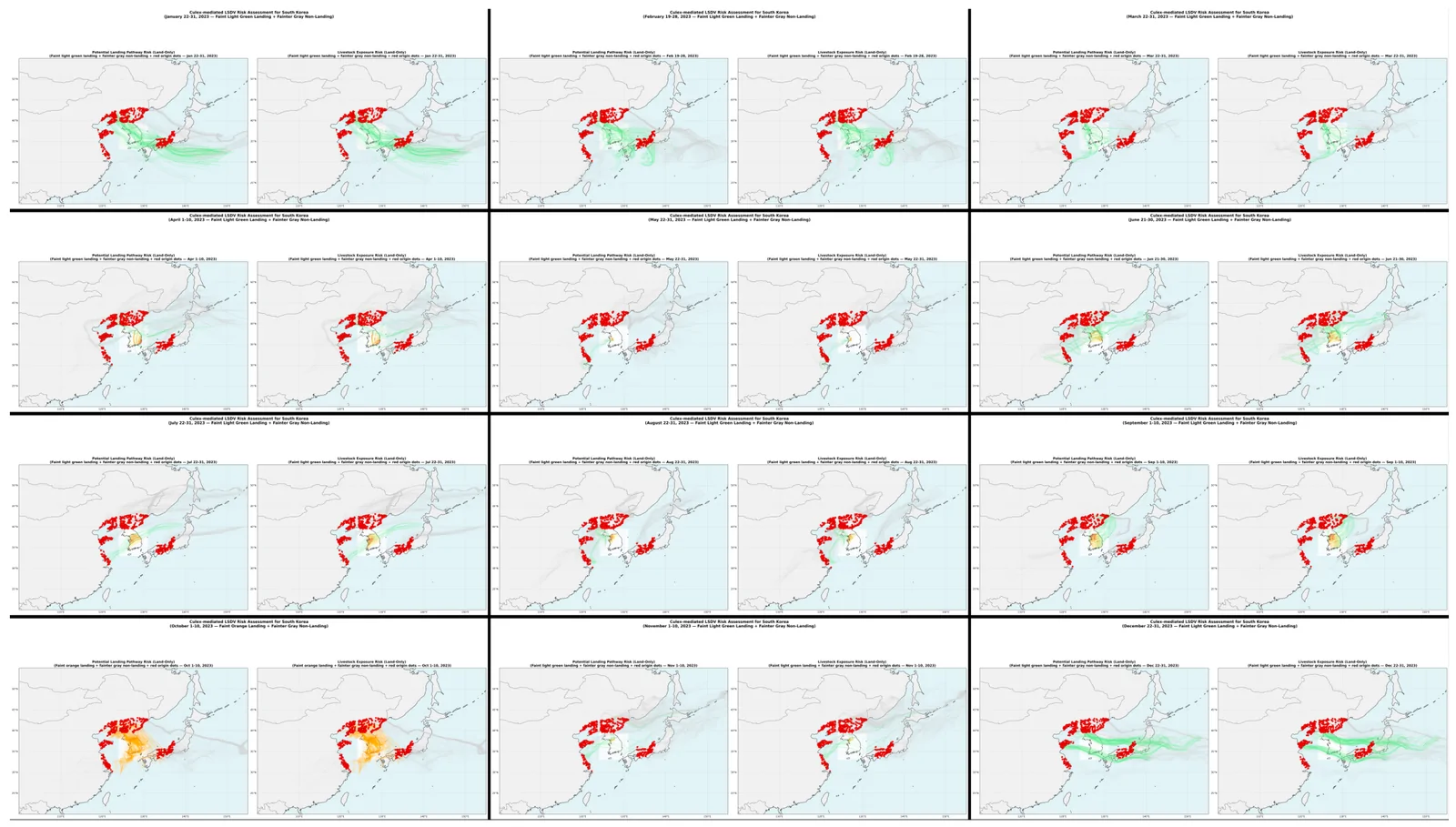
Monthly *Culex* spp.-mediated LSDV transmission risk maps for South Korea, 2023. Red dots are the origin points in the origin countries; Light green lines denote landing trajectories that reached South Korea; light grey lines denote trajectories that passed by without landing.

**Figure 7:**
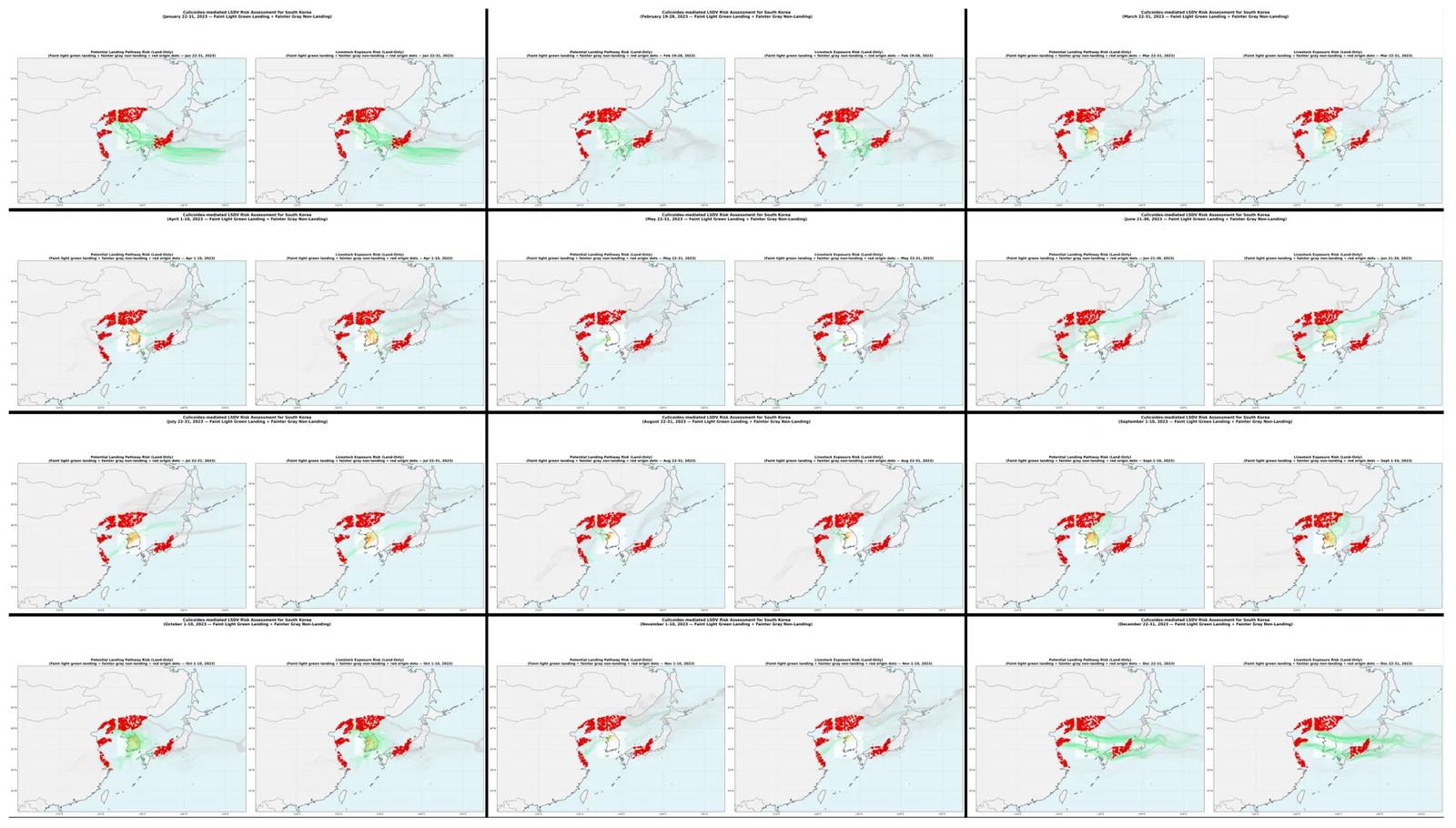
Monthly *Culicoides* spp.-mediated LSDV transmission risk maps for South Korea, 2023. Red dots are the origin points in the origin countries; Light green lines denote landing trajectories that reached South Korea; light grey lines denote trajectories that passed by without landing.

**Figure 8:**
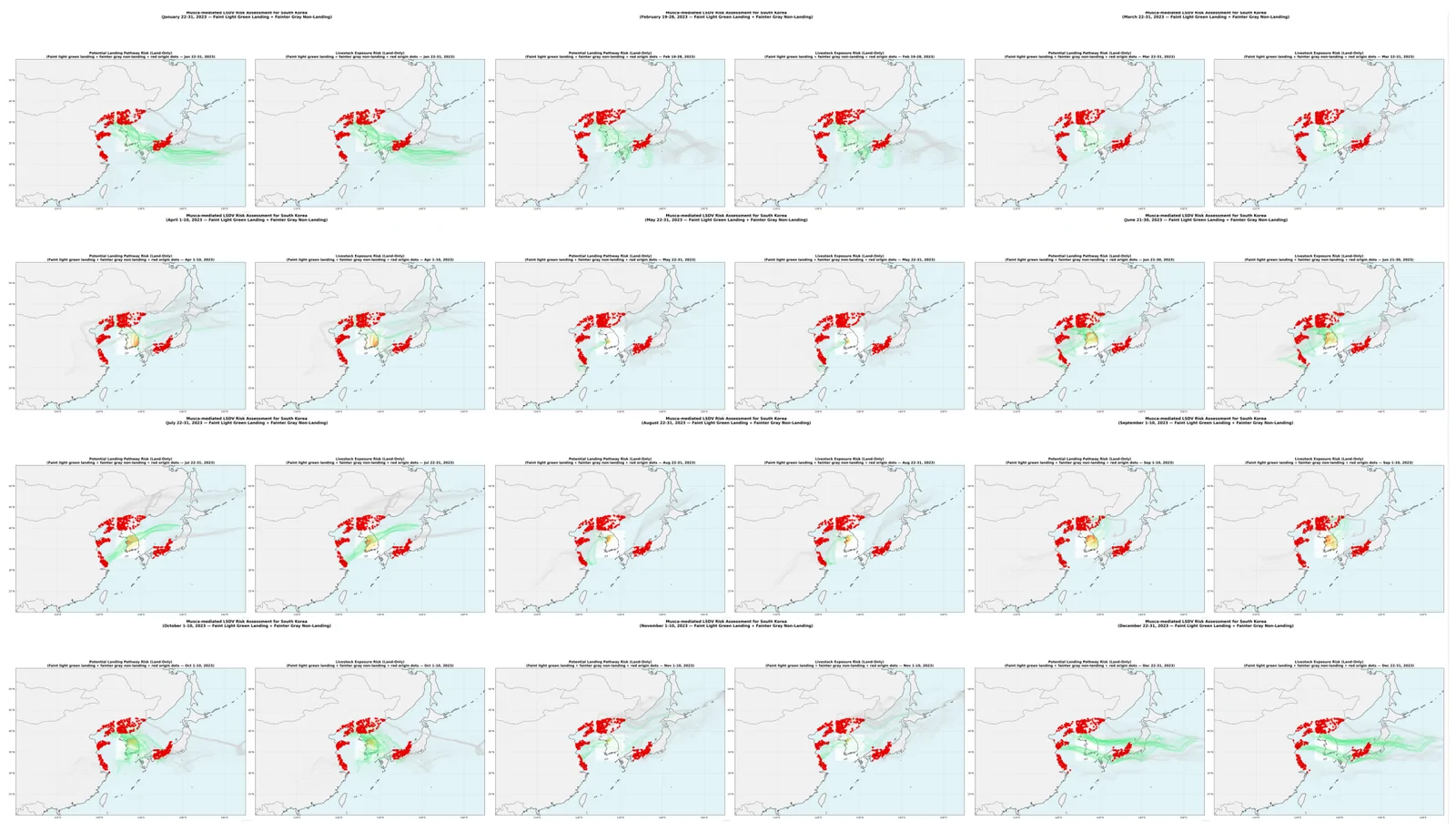
Monthly *Musca domestica*-mediated LSDV transmission risk maps for South Korea, 2023. Red dots are the origin points in the origin countries; Light green lines denote landing trajectories that reached South Korea; light grey lines denote trajectories that passed by without landing.

Of 4800 trajectories released during October 2023, 696 (14.5%) reached South Korea under settling conditions while retaining non-zero infectiousness. Successful survival-weighted landings were spatially clustered rather than uniformly distributed, with consistent concentrations along the northwestern and western coastal zones (Figure 4). Vector-specific monthly patterns are shown for *S. calcitrans* (Figure 5), *Culex* spp. (Figure 6), *Culicoides* spp. (Figure 7) and *M. domestica* (Figure 8). Post-entry inland dispersal was limited, resulting in heterogeneous exposure patterns across the country.

### 3.5. Transit Times

**Figure 9:**
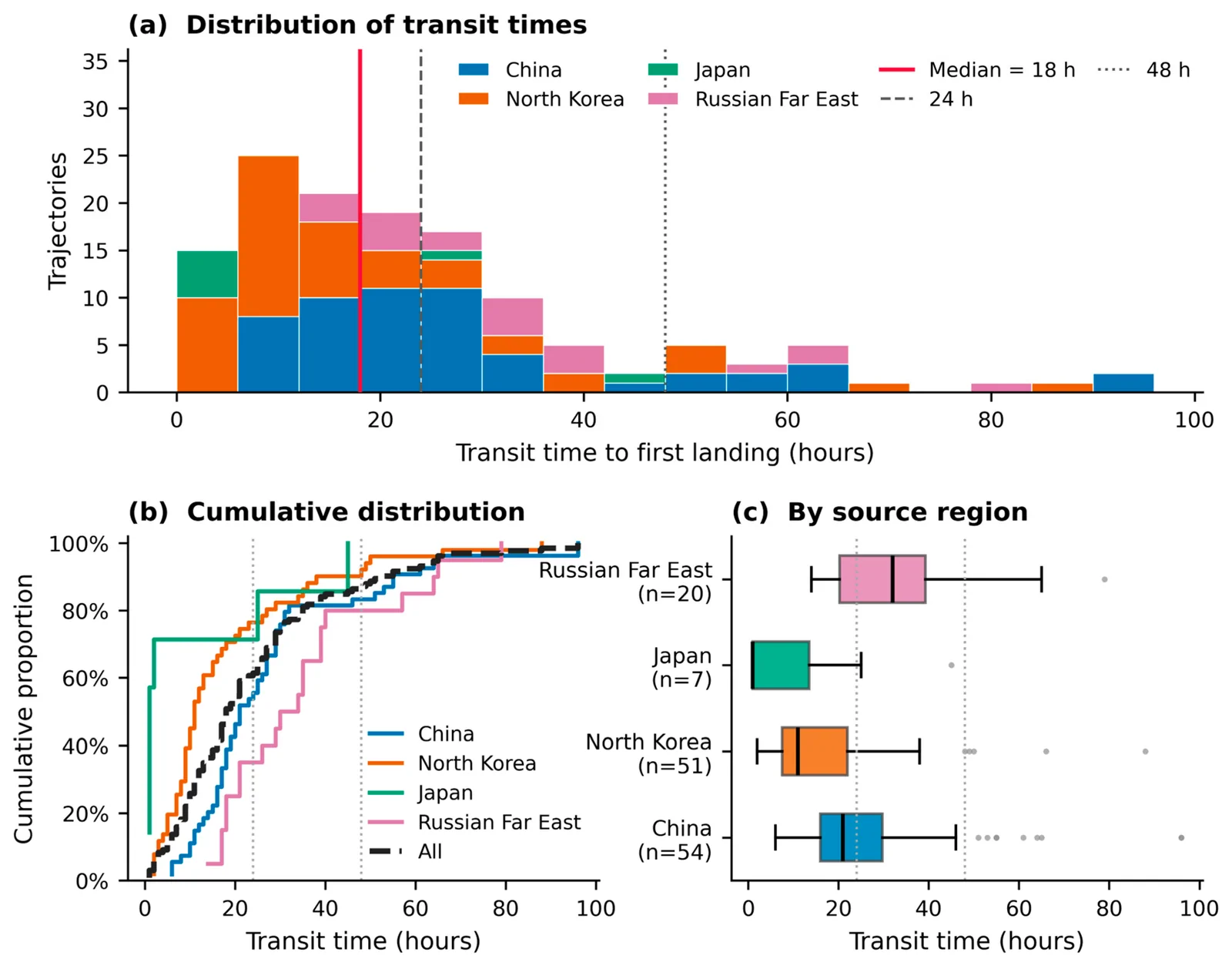
Distribution of transit times from release to first settling. (**a**) Stacked histogram by country of origin with the median and the 24 and 48 h reference points indicated. (**b**) Cumulative distribution by country of origin. (**c**) Transit time by source region (n = number of landing trajectories per region). Boxes show the interquartile range with the median as a vertical line, whiskers extend to 1.5 times the interquartile range, and grey points are outliers; dotted vertical lines mark the 24 and 48 h reference points.

From release to initial settling, the median transit duration was 18 h (interquartile range: 10.0–30.3 h; maximum: 96 h). Within 24 h, 61.4% of arrivals took place, and within 48 h, 87.1% (Figure 9, Table 3). This distribution suggests that windborne entry is possible during the infectious lifespan of a contaminated vector since mechanically delivered LSDV is kept for hours to days instead of weeks. Therefore, the retention duration is the model’s operational limitation rather than the 240 h integration ceiling.

### 3.6. Source-Region Attribution

**Figure 10:**
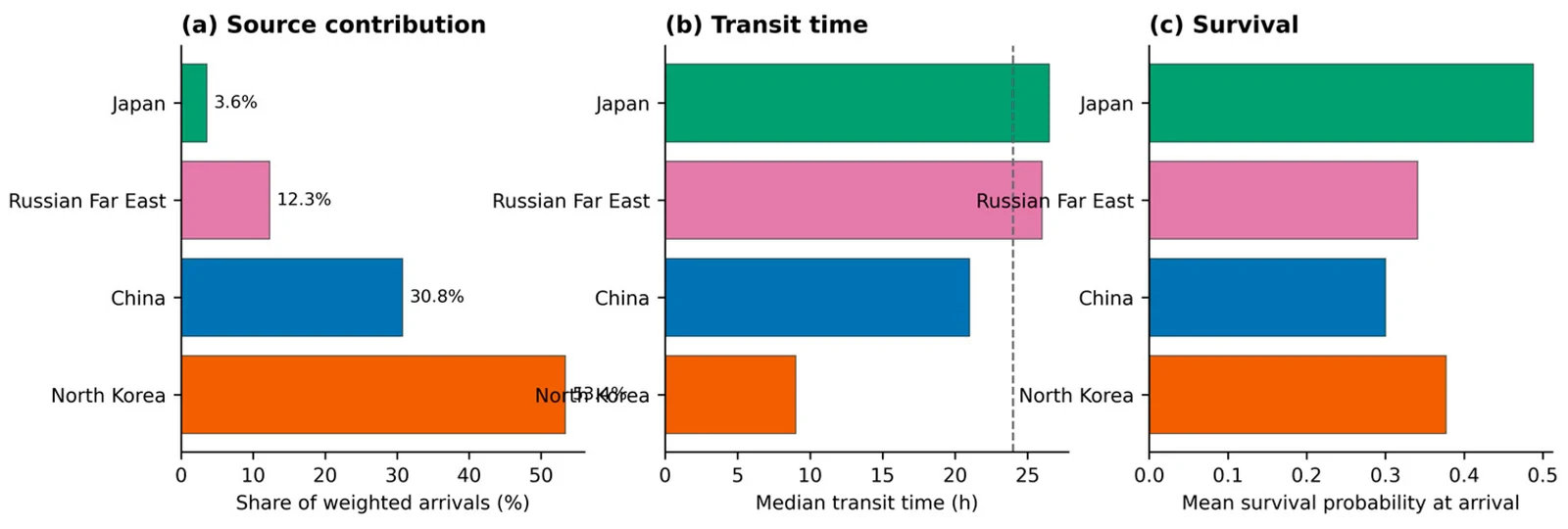
Source attribution by country of origin: (**a**) share of weighted arrival mass, (**b**) median transit time, (**c**) arrival rate as a percentage of trajectories released.

Because the sample domains span national borders, arrivals were allocated to the country in which each release cell fell rather than to the rectangular sampling area (Table 4, Figure 10). Just 100 (14.7%) of the arrived trajectories released from the Russian Far East domain originated in Russia, 295 (43.4%) in North Korea, and 263 (38.7%) in China. Of those released from the North Korea-labeled domain, China and North Korea accounted for 263 and 295 arriving trajectories, respectively.

With the shortest transit periods (median 11.0 h, IQR 7.5–22.0; 76.5% within 24 h) and the greatest arrival rate (51 arriving trajectories), North Korea accounted for 51 of the 132 arriving trajectories. With much lengthier transits (median 21.0 h, IQR 16.0–29.8; 55.6% within 24 h) and 54 arriving trajectories, China accounted for 54 of the 132 arriving trajectories. Despite seven arriving trajectories, Japan accounted for 5.3% of arriving trajectories, or an arrival rate of 71.4% within 24 h, which is in line with the dominant north-westerly flow during the fall. Russia made up 20 arriving trajectories, with 35.0% within 24 h; this figure is based on 20 arrival trajectories and should only be used as an estimate (Table 3).

Four origins could not be attributed to a country because they lay more than 25 km from any coastline in the reference boundary dataset; these were excluded from Table 4. Table 3 summarizes the minimum transit times for trajectories arriving within the 96 h virus viability window and therefore reflects fast-arrival events; Table 4 presents source attribution across the complete 240 h simulation and reflects overall cumulative exposure—the two distributions are not directly comparable.

### 3.7. Seasonal Variation in Dispersal and Exposure Risk

For each of the four vector species, the predicted infectious arrival mass peaked in September (Table 5). The arrival mass of *Culicoides* spp. decreased thirteen times between September and November, from 1.07 in September to 0.93 in October, 0.80 in August, 0.45 in July, and 0.08 in November. Because arrival mass weighs each arrival by survival and maintained infectiousness, both of which were greater under the warmer September conditions, the number of incoming trajectories peaked in October (680 of 4800; 14.2%) rather than September (616; 12.8%). The median transit time among incoming trajectories increased from 27 h in July to 37.5 h in September before decreasing to 17 h in October and 5 h in November. This reduction is not due to quicker atmospheric transport, but rather to survival selection against long-duration movement under declining temperatures.

The projected lower lethal temperature threshold was reflected in the relative ranking of vector species in October. October’s median air temperature at the 850 hPa analysis level (8.97 °C) was within the expected thresholds for *Culicoides* spp. (5 °C), *Culex* spp. (8 °C), and *S. calcitrans* and M. domestica (10 °C). This resulted in a roughly forty-fold variation in the predicted October arrival mass (0.02 to 0.93). The range was four-fold (0.25 to 1.07) in September when the mean temperature (15.9 °C) surpassed all four limits.

Predicted arrival mass is conditional on LSDV circulating in the source population. The September maximum identifies when windborne transport is most likely to succeed, given source infection.

### 3.8. Integrated Exposure Risk

Integration of survival-weighted dispersal, LSDV environmental suitability, and livestock density produced more spatially constrained exposure surfaces than dispersal alone. “High” and “Very High” exposure risk occurred only where all three components overlapped. Western and central South Korea consistently exhibited elevated exposure, particularly in areas with moderate-to-high cattle density and favorable environmental suitability.

Windborne arrival intensity and integrated exposure risk are spatially distinct. Arrival intensity is highest in the northwest, reflecting proximity to continental sources, whereas exposure risk shifts south and inland where cattle are concentrated. Across land cells, exposure risk correlated more strongly with livestock density (Spearman ρ = 0.71) than with dispersal intensity (ρ = 0.31), while dispersal intensity and livestock density were effectively independent (ρ = 0.04).

### 3.9. Sensitivity Analysis

The geographical pattern was maintained in all cases (Spearman ρ > 0.962 vs. baseline), and the predicted exposure was not affected by the humidity parameterization or a twenty-fold change in baseline mortality. Sensitivity was limited to the sole parameter that may rearrange the expected geographical pattern, the presumed lower lethal temperature threshold (ρ = 0.391) (Figure S1, Table S1). The predicted arrival mass varied by around 1.8 times over baseline mortality and by less than two times across all humidity factors.

### 3.10. Validation Using Farm-Level Data

By contrasting infected farms with 10,000 geographically independent control farms (≥30 km apart) selected among 86,120 candidate farms, the model’s performance was assessed. Compared to 34.3% of controls, 58.1% of cases in October 2023 (n = 74 infected farms) fell within “High” or “Very High” exposure risk zones (chi-square test on risk categories, *p* < 0.001). The Mann–Whitney U test on continuous exposure values indicated that the median exposure risk was 0.214 at case farms and 0.045 at control farms (*p* = 0.003).

**Figure 11:**
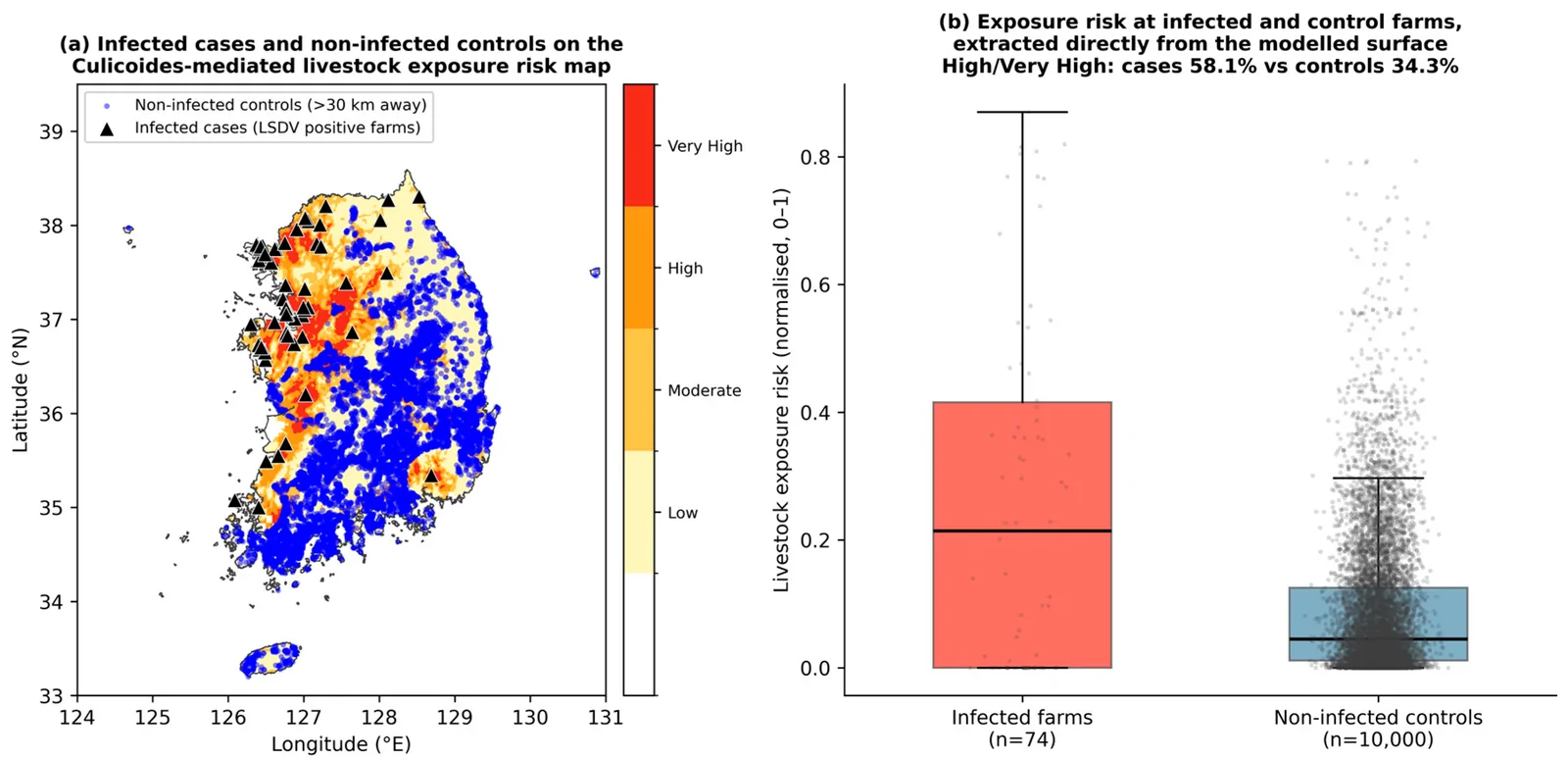
Validation of the *Culicoides*-mediated LSDV windborne dispersal risk model using a case–control design with real livestock farms. (**a**) Confirmed LSDV outbreak farms (black triangles) and non-infected control farms located at least 30 km from any case (blue dots), overlaid on the modeled livestock exposure risk map classified as low, moderate, high or very high. (**b**) Distribution of exposure risk values at case and control farm locations, extracted directly from the modelled exposure surface. Windborne transport is one of several pathways that may contribute to incursion risk, and reported outbreak locations additionally reflect subsequent local spread between farms; the comparison shown therefore provides partial rather than definitive evidence of predictive performance.

Although the difference in continuous exposure values was not statistically significant (median 0.085 vs. 0.049; Mann–Whitney U, *p* = 0.13), case farms maintained a higher representation in elevated-risk zones in November 2023 (n = 33 infected farms; chi-square *p* < 0.001). This is consistent with seasonal contraction of predicted risk. Shapiro–Wilk testing [52] supported non-parametric comparisons by confirming non-normal distributions (*p* < 0.001 for both groups in both months). Infected farms were found to preferentially cluster inside or close to predicted high-risk zones in the northwest and west, while control farms were distributed throughout lower-risk areas, according to spatial visualization (Figure 11). As a result, during the peak transmission period, the model distinguished between case and control farms, with less discrimination during late-season circumstances.

Farm-level epidemic sites should be taken into consideration when interpreting this connection. The evaluation provided here is pathway-specific rather than an estimate of overall introduction risk, and windborne dispersal is one of numerous paths that may contribute to LSDV invasion, along with live animal movement, animal products, fomites, and vector-laden conveyance (Section 4.5). The elevated exposure seen at case farms is consistent with both windborne introduction and local transmission occurring within areas that are also environmentally suitable for vector activity, as reported outbreak locations also reflect the point of introduction and subsequent local spread between neighboring farms. Therefore, without clarifying the proportional impact of the two processes, the comparison shows that outbreaks occurred disproportionately inside modeled high-risk locations.

Additionally, two aspects of the design are worth mentioning. Livestock density, which is higher in regions with more farms, is taken into account in the exposure index. Additionally, controls had to be at least 30 km away from any case, excluding the farms that most closely resembled cases. Sensitivity analyses (Section 2.6.1) and spatial permutation tests were used to evaluate resilience apart from these features. The dispersal component’s observed likelihood-ratio statistic was exceeded in 23.6% of 499 permutations (χ^2^ = 10.4, empirical *p* = 0.236; Figure S2, Table 6) and the concentration of outbreaks within the highest exposure quartile in 17.2% when the dispersal surface was randomly translated throughout the study area.

The 74 outbreaks that were reported took place between October 19 and October 31, 2023, a span of 13 days. As predicted of a spreading epidemic rather than simultaneous separate introductions, pairwise spatial and temporal distances between cases were positively linked (Mantel r = +0.136, permutation *p* = 0.013; Figure S3), meaning that cases that were further away geographically were also farther apart in time. The outbreaks were divided into 25 clusters by spatiotemporal grouping (15 km, 14 days); six of these clusters had several farms and together accounted for 55 cases; the four largest clusters had 49 farms (Figure S4). Furthermore, 25 should be considered an upper bound on the number of separate introduction events, as some single-farm clusters can indicate undiscovered local spread. This trend suggests that epidemic sites at the farm level are not statistically independent observations.

Complementary questions are addressed in Figures S3 and S4: Figure S3 illustrates whether outbreaks are clustered into transmission clusters, and Figure S4 illustrates whether these clusters spread geographically as the epidemic worsened. When combined, they show that the observed epidemic map represents both the initial introduction and the continuing local transmission, not only the introduction.

When combined, these analyses show that more distant farms were infected at various periods, whereas close outbreak farms tended to become infected at comparable times. This is a true but weak connection (Mantel r = +0.136) that is unlikely to have occurred by chance (permutation *p* = 0.013). Rather than being distinct arrivals, the majority of reported outbreaks were part of a limited number of transmission groups: six of the 25 spatiotemporal clusters found included 55 of the 74 impacted farms (Figure S4). After the virus entered South Korea, the epidemic expanded from farm to farm, according to the epidemiological reading. As a result, recorded outbreak locations document both the place of introduction and subsequent local transmission.

The permutation test revealed that comparable agreement could be achieved in 23.6% of randomly shifted maps, despite the fact that case farms were situated in regions with greater anticipated windborne exposure. This suggests that a pathway-specific introduction model can only be partially validated by outbreak sites alone, as subsequent local farm-to-farm transmission also has a significant impact on the observed epidemic pattern.

## 4. Discussion

This study estimated habitat suitability for four major LSDV-associated vector species using climatic and topographic predictors and integrated these outputs with livestock density to generate spatially explicit maps of LSD outbreak and windborne exposure risks in South Korea.

*Culex* spp. showed high suitability along the western Korean coast and eastern Chinese coast, driven primarily by NDVI and elevation. This aligns with the ecological preference of *Culex tritaeniorhynchus* for vegetated, sunlit aquatic habitats such as rice paddies [53] and with established positive relationships between NDVI and mosquito abundance and diversity [54]. *M. domestica* exhibited a latitudinal gradient, with suitability increasing southward, driven by wind speed, mean temperature of the wettest quarter, and solar radiation. This reflects the temperature dependence of *M. domestica* activity [55,56] and the role of warm, solar-irradiated environments in accelerating manure decomposition, which serves as a key larval substrate in southern livestock systems [57]. *S. calcitrans* showed high suitability in coastal areas, with elevation emerging as the dominant predictor (99.4%). Elevation likely serves as a proxy for low-lying, intensively managed livestock and agricultural settings that support larval development in manure-rich, moist substrates [58,59,60]. *Culicoides* spp. exhibited the most spatially uniform distribution, attributable to the genus’s species diversity (~1400 species) and their capacity to breed across diverse semi-aquatic habitats [61,62]. NDVI and precipitation were the dominant predictors, reflecting soil moisture and microbial food availability for larvae [62].

LSD outbreak risk was highest along the coasts of North Korea, China, and Russia, with the majority of areas in Japan demonstrating low risk. This spatial pattern is consistent with the epidemiology of LSD introduction in East Asia, where early outbreaks were frequently reported in coastal areas [8,63,64], including South Korea’s first confirmed case [65].

The inclusion of multiple vector species is supported by experimental and field evidence indicating that blood-feeding Diptera, including stable flies, mosquitoes, and biting midges, can acquire and mechanically retain LSDV after contact with infectious lesions, with viral retention on mouthparts enabling transmission during interrupted feeding [42,66]. Mechanistic models suggest that *S. calcitrans* exhibit the highest transmission efficiency among common vectors, although multiple species contribute to LSDV transmission under field conditions [46]. Importantly, mechanical transmission efficiency depends on viral load in donor lesions and vector biting frequency [24], which may explain why exposure risk does not always translate to outbreaks.

### 4.1. Feasibility and Seasonal Structure of Windborne Introduction

Windborne dispersal modeling that integrates vector survival, retained infectiousness, LSDV environmental suitability, and livestock density revealed that arrival from continental sources is feasible within the infectious lifetime of a mechanically contaminated vector. As the median air temperature at the analysis level dropped from 15.9 °C to 2.7 °C, the predicted arrival mass peaked in September and decreased thirteen times by November. This is in line with field data showing LSD incidence clusters during warm seasons, the temperature-dependent activity of biting arthropods, and the documented inefficiency of mechanical transmission in cold and dry environments [16]. Windborne LSDV introduction into Israel from Egypt has previously been studied using similar trajectory-based analyses [67]. Comparable frameworks have been applied to bluetongue virus incursion into mainland Europe from Sardinia [20] and to *Culicoides* incursions into northern Australia [68].

### 4.2. Independent Corroboration from the National Epidemiological Investigation

The official epidemiological examination of the 2023 Korean outbreak undertaken by the Animal and Plant Quarantine Agency [69] provides an independent test of the seasonal window outlined here. In order to determine air currents that may carry haematophagous insects from mainland China to the west coast of Korea, the study used a single-particle Lagrangian dispersion model (NOAA HYSPLIT). It predicted the introduction time as being before 20 September 2023. Westerly airflow appropriate for such conveyance was discovered on 18–19 September and 27–30 September, with the Shandong Peninsula identified as the nearest probable source at around 316 km from the Korean west coast.

There are three noteworthy points of agreement. First, according to our model, all four vector species have their maximum infectious arrival mass in September (Table 5), which coincides with the official estimated introduction date. Second, using an incubation period of 4–14 days and a reporting delay of 2–10 days, back calculation from the first case reported on 19 October 2023 places introduction between 25 September and 13 October, covering the latter of the two indicated airflow windows. Third, at the wind speeds simulated here, our source attribution assigns 22.7% of weighted arrivals to China at a median transit of 25 h, which is consistent with a 316 km passage. These correlations result from different data and methods: the current work employed forward Runge–Kutta integration from suitability-weighted source cells with explicit survival and viral retention weighting, while the official analysis used backward HYSPLIT trajectories from receptor sites.

Additionally, 21,897 mosquitoes and biting midges collected at 32 monitoring locations between May and October 2023 proved negative for LSDV, according to the national inquiry [69]. This does not refute windborne introduction because the estimated number of positive vectors in a sample of that size is significantly less than one, given a source infection frequency on the order of 10^−3^ (Section 2.5) and the short retention of mechanically delivered virus. It does, however, show why mechanistic evaluation of the type described here is necessary and why entomological surveillance is unlikely to directly corroborate this pathway.

Significantly, the same study noted 3308 vessel landings from thirteen LSD-affected nations at seven major Korean ports in September and October 2023 [69], identifying vessel-borne importation of infected vectors as a competing explanation. This supports the idea that windborne dispersal should be evaluated as one contributor among several rather than as the exclusive route of entrance and is consistent with the vector-laden transport pathway examined in Section 3.4 and Table 7.

There are more examples of LSDV being introduced by wind. Backward Lagrangian trajectory analysis revealed atmospheric connections between impacted Egyptian localities and the Israeli receptor sites during the pertinent synoptic systems, and the Israeli outbreaks of 1989 and 2006 each coincided with severe epizootics in Egypt during a period when cattle imports were strictly regulated [67]. An arthropod-mediated rather than contact-mediated method of transmission was further substantiated by mathematical modeling of the 2006 Israeli outbreak [70]. Independent reports have demonstrated the long-distance wind-assisted spread of *Culicoides* spp. into Israel [72]. Although it is shorter than the Yellow Sea crossing, wind-driven displacement of *S. calcitrans* up to 225 km behind weather fronts has been documented [73], indicating that passive movement of this species occurs at a scale of hundreds of kilometers.

### 4.3. Vector Competence and Dispersal Capacity

An important qualification of any windborne assessment of LSDV is that the two components of the pathway are supported by evidence from different species. Passive wind-assisted transport over hundreds of kilometers is documented for *Culicoides* spp. [22,23], which is why this genus was treated as the primary vector here. Experimental transmission of LSDV by *C. nubeculosus* has nevertheless not been achieved [24], despite this species acquiring and retaining virus and being assigned the second-highest transmission potential of four model vectors [21]. Conversely, *S. calcitrans* has the highest experimentally derived transmission potential [21,24] and demonstrated mechanical transmission, but evidence for passive long-distance transport is limited.

Windborne introduction of LSDV therefore requires either that *Culicoides* spp. transmit under field conditions in a manner not yet reproduced experimentally, or that species with demonstrated competence are transported further than is currently documented. The present results should accordingly be read as quantifying the atmospheric component of this pathway; the biological component remains incompletely characterized. The retention constant and source infection prevalence applied in Section 2.4.7 are derived from experimental studies of related vector species rather than from field data for *Culicoides* spp. in this setting, and both were varied across their plausible ranges in sensitivity analysis (Section 2.6.1).

### 4.4. Implications for Surveillance

These findings have important implications for risk-based planning. Intensified surveillance, vector control, and livestock biosecurity measures should be prioritized during the periods of highest predicted arrival mass, when environmental suitability and vector dispersal overlap most strongly. Integrated vector management, including reduction in arthropod breeding sites, application of repellents, and targeted vector control around livestock facilities, is required during this window. The potential role of sub-clinically infected cattle in maintaining low-level viral circulation further underscores the need for surveillance extending beyond clinically apparent cases [66]. Because the model does not predict which individual farms or districts will experience outbreaks, its appropriate application is to the timing and source-region targeting of surveillance rather than to the geographic prioritization of individual holdings.

The limited spatial specificity of the exposure surface has a direct bearing on how it should be used. Because reported outbreak locations are dominated by post-introduction farm-to-farm spread, the surface should not be used to prioritize individual holdings for inspection, movement restriction or pre-emptive vaccination. It does, however, support three applications that do not depend on farm-level specificity: timing the intensification of vector surveillance to the predicted seasonal window, which peaked in September across all four vector species; concentrating entomological sampling in the northwestern and western coastal districts during that window; and prioritizing diagnostic follow-up of source-region intelligence, particularly from the northern land border, where predicted transit times are shortest at a median of 9.5 h.

Because the framework does not identify which individual holdings will experience outbreaks, its appropriate application is to the timing and source-region targeting of surveillance rather than to the geographic prioritization of individual farms. It characterizes the plausibility and seasonal structure of windborne introduction and does not establish windborne transport as the source of the 2023 Korean epidemic.

### 4.5. Windborne Transport Among Other Introduction Pathways

There are several ways to introduce LSDV into a free region, and an evaluation limited to one pathway characterizes a portion of risk rather than the entirety of it (Table 7). The virus’s gradual spread through the Middle East, the Balkans, and eventually Asia has been linked to the movement of live animals, which is typically thought to be the predominant route at the continental scale [4,74]. The WOAH Terrestrial Animal Health Code’s import guidelines for LSD-free nations [71] limit this route for the Republic of Korea; nonetheless, unreported transit across the land border with the Democratic People’s Republic of Korea cannot be ruled out.

The use of animal products is a reasonable but inadequately measured approach. Long-term persistence of LSDV in bovine semen has been shown in experiments [74], with live virus recovered up to 42 days and viral DNA up to 159 days post-infection [75]. Seminal transmission to heifers has also been shown. Additionally, the virus has been found in skin lesions, saliva, milk, and nasal secretions [74]. Therefore, regardless of vector activity or season, artificial insemination and the global exchange of bovine genetic material form a unique introduction mechanism.

Although their involvement in international introduction is less well defined, contaminated vehicles, equipment, and personnel have been related to the transmission across holdings during outbreaks [76]. For haematophagous Diptera, vector-laden transport—in which infected arthropods are transported passively aboard vehicles or vessels rather than by wind—is recognized [14] and is independent of weather; stable flies can also contaminate the environment through regurgitation and defecation [77], prolonging the time that a transported vector poses a risk. Compared to other capripoxviruses, wildlife seems to have a lesser role since there is no known reservoir that can sustain or spread the virus over great distances [74].

Unlike all of the aforementioned, the windborne route modeled in this study is entirely regulated by vector biology and weather, making it both highly seasonal and theoretically predictable. Additionally, the biological evidence for this route is the least comprehensive; the relative roles of biting midges and mosquitoes as mechanical LSDV vectors are still unknown [12]. It was outside the purview of this study to conduct a comparative import risk analysis that integrated trade volumes, consignment-level contamination probability, and border control efficacy in order to quantify the proportional impact of each channel.

## 5. Limitations

First, and most importantly, the association between predicted exposure and observed outbreak locations was not robust to spatial permutation: randomly relocating the dispersal surface reproduced comparable agreement in 23.6% of permutations. The framework therefore characterizes the plausibility and seasonal structure of windborne introduction and does not establish windborne transport as the source of the 2023 Korean epidemic. Second, experimental transmission of LSDV by *Culicoides* spp., the primary vector modeled here, has not been demonstrated, and no genomic data were available to link reported outbreaks to distinct introduction events (Section 4.3).

Third, source infection prevalence and virus retention parameters were derived from experimental studies of related vector species rather than from field data for *Culicoides* spp. in this setting, and no term for the probability of vector uplift into a Windstream was included. Fourth, thermal survival was evaluated using air temperature at 850 hPa (~1458 m), which is colder than the near-surface conditions for which arthropod thermal tolerances are established. A lapse-rate correction was applied as a sensitivity scenario, and sensitivity analysis identified this threshold as the principal source of parametric uncertainty. Fifth, vector occurrence records were obtained from an opportunistic biodiversity database and are subject to sampling bias. Two of the four resulting suitability surfaces were dominated by a single covariate and should be regarded as preliminary. Sixth, the driving reanalysis has a horizontal resolution of 0.25°, which sets the finest scale at which the dispersal component can be resolved, and landing counts varied by approximately 18% between runs under identical settings. Seventh, the analysis covered July to November 2023. Predicted arrival mass peaked in September within this window; months outside it were not modeled. The model does not explicitly simulate vector breeding dynamics, and uncertainty in species-specific transmission efficiency may influence the exposure surface.

## 6. Conclusions

This study developed a mechanistic framework for assessing windborne introduction of LSDV into South Korea and applied it to July–November 2023. Simulated arrival was feasible within the infectious lifetime of a mechanically contaminated vector, with a median transit of 18 h and 87.1% of arrivals occurring within 48 h. Predicted infectious arrival mass peaked in September across all four vector species and declined thirteen-fold by November. North Korea contributed 76.5% of weighted arrivals at the shortest median transit of 11.0 h, with China contributing 55.6% at 21.0 h median.

Comparison against the October 2023 epidemic showed that infected farms occupied areas of higher predicted exposure than non-infected controls, but this association was not robust to spatial permutation. Because reported outbreak locations reflect both the point of introduction and subsequent local transmission, they provide only limited validation of a pathway-specific introduction model. The framework is therefore suited to informing the timing and source-region focus of vector surveillance rather than the identification of individual at-risk holdings, and windborne transport should be regarded as one of several pathways that may contribute to incursion risk.

## Figures and Tables

**Table 1:** Summary of bioclimatic and environmental variables used for MaxEnt model prediction.

Variables	Signification	Time Scale	Spatial Resolution	Data Source
*Culex*	*Culex* spp. occurrence coordinates	2000–2025	NA	GBIF
*Musca*	*Musca* spp. occurrence coordinates	2000–2025	NA	GBIF
*Stomo*	*Stomoxys calcitrans* occurrence coordinates	2000–2025	NA	GBIF
*Culico*	*Culicodes* spp. occurrence coordinates	2000–2025	NA	GBIF
LSD	Lumpy skin disease occurrence coordinates	1 January 2006–5 November 2025	NA	FAO Empres-i
bio1	Annual mean temperature	1970–2000	1 × 1 km	WorldClim 2.1
bio2	Mean diurnal range	1970–2000	1 × 1 km	WorldClim 2.1
bio3	Isothermality (bio2/bio7) (×100)	1970–2000	1 × 1 km	WorldClim 2.1
bio4	Temperature seasonality (standard deviation × 100)	1970–2000	1 × 1 km	WorldClim 2.1
bio5	Max temperature of the warmest month	1970–2000	1 × 1 km	WorldClim 2.1
bio6	Min temperature of the coldest month	1970–2000	1 × 1 km	WorldClim 2.1
bio7	Temperature annual range (bio5–bio6)	1970–2000	1 × 1 km	WorldClim 2.1
bio8	Mean temperature of the wettest quarter	1970–2000	1 × 1 km	WorldClim 2.1
bio9	Mean temperature of the driest quarter	1970–2000	1 × 1 km	WorldClim 2.1
bio10	Mean temperature of the warmest quarter	1970–2000	1 × 1 km	WorldClim 2.1
bio11	Mean temperature of the coldest quarter	1970–2000	1 × 1 km	WorldClim 2.1
bio12	Annual precipitation	1970–2000	1 × 1 km	WorldClim 2.1
bio13	Precipitation of the wettest month	1970–2000	1 × 1 km	WorldClim 2.1
bio14	Mean precipitation of the driest month	1970–2000	1 × 1 km	WorldClim 2.1
bio15	Precipitation seasonality	1970–2000	1 × 1 km	WorldClim 2.1
bio16	Precipitation of the wettest quarter	1970–2000	1 × 1 km	WorldClim 2.1
bio17	Precipitation of the driest quarter	1970–2000	1 × 1 km	WorldClim 2.1
bio18	Mean precipitation of the warmest quarter	1970–2000	1 × 1 km	WorldClim 2.1
bio19	Mean precipitation of the coldest quarter	1970–2000	1 × 1 km	WorldClim 2.1
srad	Solar radiation	1970–2000	1 × 1 km	WorldClim 2.1
wind	Wind speed	1970–2000	1 × 1 km	WorldClim 2.1
elev	Elevation	1970–2000	1 × 1 km	WorldClim 2.1
NDVI	Normalized Difference Vegetation Index	2015–2024	1 × 1 km	NASA EarthData
cattle	Cattle density	2015	10 × 10 km	FAO livestock
buffalo	Buffalo density	2015	10 × 10 km	FAO livestock

**Table 2:** Model configuration and parameter values for the windborne transport simulation. Parameters derived from experimental literature are identified as such; those not empirically constrained were varied in sensitivity analysis.

Parameter	Value
Trajectories per month	4800
Origins per source region	1200
Maximum integration time	240 h
Timestep	1.0 h
Integration scheme	Runge–Kutta 4th order
Wind field	ERA5, 850 hPa (~1458 m), 0.25°
Unit conversion	3600·v/111,320 (deg h^−1^ per m s^−1^)
Source region buffer	500 km from Korean land border
Smoothing bandwidth	25 km (matched to ERA5 native)
Output grid	0.009° (~1 km), reported at 25 km/district
Source infection prevalence	0.001
Virus retention time constant	48 h
Maximum infectious duration	96 h
Lower lethal temperature	5 °C
Upper lethal temperature	40 °C
Baseline hourly mortality	0.01
Random seed	42 (fixed for reproducibility)
Simulation period	Full calendar year 2023 (12 monthly runs)

**Table 3:** Minimum transit times for arriving trajectories (≤96 h viability window), by source country. Values are medians with interquartile ranges; percentages indicate the proportion of arrivals occurring within 24 and 48 h with maximum 96 h.

Source Region	Arriving Trajectories (n)	Median Transit Time (h)	Interquartile Range (h)	Maximum Transit Time (h)	Arrivals Within 24 h (%)	Arrivals Within 48 h (%)
China	54	21.0	16.0–29.8	96	55.6	83.3
North Korea	51	11.0	7.5–22.0	88	76.5	92.2
Japan	7	1.0	1.0–13.5	45	71.4	100.0
Russian Far East	20	32.0	20.3–39.3	79	35.0	80.0
All regions	132	18.0	10.0–30.3	96	61.4	87.1

**Table 4:** Source attribution across all arriving trajectories (full 240 h simulation), by source country: trajectories released, arrival rate, share of weighted arrival mass, median transit time and mean survival probability at arrival. Arrivals are attributed to the country in which each release cell falls rather than to the rectangular sampling domain.

Source Region	Arriving Trajectories (n)	Share of Weighted Arrival Mass (%)	Median Transit Time (h)	Mean Survival Probability at Arrival
China	263	0.304	21	0.28
Japan	22	0.035	26.5	0.45
NorthKorea	295	0.538	9	0.36
RussiaFE	100	0.122	25.5	0.32

**Table 5:** Seasonal comparison of arrival metrics across July to November 2023, by vector species.

Vector Species	Month	Trajectories Released (n)	Arriving Trajectories (n)	Arrival Rate (% of Trajectories Released)	Median Transit Time (h)	Total Weighted Arrival Mass	Mean Exposure Risk over Land (Normalized, 0–1)	Median Air Temperature at 850 hPa (°C)
*Culicoides* spp.	July	4800	348	7.25	27.00	0.449	0.0595	17.95
	August	4800	574	11.96	33.00	0.803	0.0426	18.02
	September	4800	616	12.83	37.50	1.066	0.0656	15.89
	October	4800	680	14.17	17.00	0.931	0.0725	8.97
	November	4800	254	5.29	5.00	0.079	0.0373	2.68
*Culex* spp.	July	4800	356	7.42	24.00	0.350	0.0648	17.95
	August	4800	477	9.94	29.00	0.483	0.0589	18.02
	September	4800	518	10.79	32.00	0.561	0.0687	15.89
	October	4800	292	6.08	6.00	0.159	0.0555	8.97
	November	4800	299	6.23	4.00	0.036	0.0244	2.68
*Stomoxys calcitrans*	July	4800	350	7.29	23.50	0.338	0.0582	17.95
	August	4800	465	9.69	28.00	0.471	0.0579	18.02
	September	4800	489	10.19	34.00	0.519	0.0635	15.89
	October	4800	201	4.19	5.00	0.022	0.0423	8.97
	November	4800	262	5.46	5.00	0.027	0.0176	2.68
*Musca domestica*	July	4800	353	7.35	21.00	0.219	0.0492	17.95
	August	4800	394	8.21	23.00	0.233	0.0505	18.02
	September	4800	396	8.25	29.00	0.252	0.0472	15.89
	October	4800	235	4.90	4.00	0.024	0.0405	8.97
	November	4800	302	6.29	4.00	0.012	0.0271	2.68

“Equal allocation across source regions is a stratified sampling design intended to provide comparable statistical power for each region; all inter-regional comparisons therefore use arrival rate (proportion of released trajectories resulting in arrival with viable virus) rather than arrival count”.

**Table 6:** Validation of the windborne dispersal component: observed likelihood-ratio statistic, asymptotic and permutation-based *p*-values, and the null distribution obtained under random spatial translation of the dispersal surface.

Test Statistic	Observed Likelihood-Ratio Statistic (χ^2^)	Asymptotic *p*-Value (χ^2^, 1 df)	Permutation *p*-Value (499 Random Translations)	Mean χ^2^ Under Random Translation	95th Percentile of Null χ^2^ Distribution	Districts Included (n)	Outbreaks Included (n)
Nested negative binomial likelihood-ratio test for the log dispersal surface	10.425	0.001	0.236	6.840	25.481	196	74

**Table 7:** Potential routes of LSDV introduction into South Korea: mechanism, relevance to the Republic of Korea, seasonality, and whether quantified in this study.

Introduction Pathway	Transmission Mechanism	Relevance to the Republic of Korea	Seasonality	Relative Importance for South Korea	Quantification Status	Key References
Movement of live animals	Transport of viraemic cattle across international borders	Constrained by WOAH import recommendations for LSD-free countries; unrecorded movement across the land border with the DPRK cannot be excluded	None	Low—legal cattle imports are restricted; the DPRK land border is the principal residual uncertainty but is unquantified	Not quantified; assessed qualitatively against trade and import data	[4,70]
Trade in animal products	LSDV remains viable in hides and has been detected in milk, saliva, nasal secretions and skin lesions	Plausible; probability per consignment low but trade volume substantial	None	Low–moderate—individual consignment risk is low; aggregate risk scales with import volume from affected regions	Not quantified in this study	[70]
Semen and bovine genetic material	Live virus recovered from bull semen to 42 days and viral DNA to 159 days post-infection; seminal transmission demonstrated experimentally	Documented pathway; Korea imports bovine genetic material internationally	None	Moderate—active and ongoing import pathway; long viral persistence makes this uniquely independent of season or vector activity	Not quantified in this study	[44,69]
Fomites and vehicles	Contaminated transport vehicles, equipment, feed and personnel	Established for farm-to-farm spread during epidemics; role in international introduction less well characterized	Weak	Low for index introduction—primarily relevant to within-country amplification once the virus is established	Not quantified in this study	[70]
Vector-laden transport	Infected haematophagous Diptera carried passively aboard vehicles, vessels or aircraft; stable flies additionally contaminate the environment through regurgitation and defecation	Plausible given port and air connectivity with continental Asia; the national investigation recorded 3308 vessel arrivals from 13 LSD-affected countries at seven major Korean ports during September and October 2023	Weak	Moderate–High—independently identified as a competing hypothesis of comparable plausibility in the 2023 national epidemiological investigation; not weather-dependent, making it active year-round	Not quantified in this study; ranked co-equal with windborne route by the national investigation [69]	[14,48,49]
Wildlife	Movement of infected wild ruminants	No established reservoir maintaining or translocating the virus over long distances; competence of Hydropotes inermis unknown	Unknown	Unknown—absence of a confirmed sylvatic reservoir limits assessment; cannot be excluded as a secondary amplifying host	Not quantified; insufficient field data	[71]
Windborne vectors	Passive wind-assisted dispersal of infected Diptera within the atmospheric boundary layer	Meteorologically feasible; median simulated transit 17 h, with 87% of arrivals within 48 h. Precedent established for LSDV introduction into Israel from Egypt	Strong	Moderate—seasonally concentrated and independently corroborated by HYSPLIT analysis; spatial association with 2023 outbreak farms is present but not robust to permutation (23.6%), indicating this route cannot be confirmed as the sole or dominant source	Quantified—this study; forward RK4 trajectory integration with survival and virus-retention weighting	[23,25,67,68]

Abbreviation: DPRK: Democratic People’s Republic of Korea (North Korea).

## Data Availability

ERA5 reanalysis data are available from the Copernicus Climate Data Store. Vector occurrence records are available from GBIF and LSD occurrence records from FAO EMPRES-i. Analysis code is available upon request. Supplementary Materials comprises Table S1 and Figures S1–S4 as listed above.

## Supplementary Materials

The following supporting information can be downloaded at: https://www.mdpi.com/article/10.3390/ani16182866/s1, Figure S1: Sensitivity of predicted arrival mass to survival parameters; Figure S2: Null distribution of the likelihood-ratio statistic; Figure S3: Space-time structure of the October 2023 outbreaks; Figure S4: Spatio-temporal clustering of October 2023 LSD outbreaks; Table S1: Sensitivity Analysis; Word document: Mathematical Model Runge Kutta RK4 for Wind borne Transmission.
