# Supplementary material for: Windborne Dispersal of Arthropod Vectors as a Pathway for Lumpy Skin Disease Virus Introduction into South Korea: A Multi-Species, Source-Attributed Modelling Study
Source: Animals (Basel). 2026 Sep 11;16(18):2866. doi: 10.3390/ani16182866 (PMC13603381; doi:10.3390/ani16182866)
Supplement: Supplementary file 1 [file animals-16-02866-s001.zip › Mathematical Model Runge Kutta RK4 for Wind borne Transmission.pdf]

Mathematical Framework for the Runge-Kutta (RK4) Windborne Transport Model for LSDV (Vectors-Specific Implementation)

The Step-wise revised mathematical model parameters are given;

Maximum simulation time: 240 hours (10 days).

Time step Δt: 1/2 hour.

Survival: Hourly hazard-based multiplicative survival (hard cutoffs + quadratic penalties for T, RH, wind stress).

Landing condition: Trajectory enters strict South Korea land polygon **and** has downward vertical velocity (w > 0) at that step.

Dispersal risk: Survival-weighted accumulation of **all positions inside Korea** (not just first arrival or final).

Exposure risk: Dispersal × LSDV susceptibility × livestock density.

Smoothing: Gaussian filter (σ ≈ 3 km).

Risk classification: Percentile-based (50th, 75th, 90th on non-zero cells).

1. Model Domain and Variables

Let the position of a particle (insect) at time t(hours) be:

$$\mathbf{X}(t) = [\phi(t), \lambda(t)]$$

where:

- ϕ= latitude (°N)
- λ= longitude (°E)

The wind field is interpolated from ERA5 surface level (index 0):

$$\mathbf{V}(t, \phi, \lambda) = [u(t, \phi, \lambda), v(t, \phi, \lambda), w(t, \phi, \lambda)]$$

where:

- u= zonal wind component (m s^{-1})
- v= meridional wind component (m s^{-1})
- w= vertical velocity (m s^{-1}, positive downward in ERA5 convention)

Additional fields:

- $T(t, \phi, \lambda)$= temperature (K)
- $RH(t, \phi, \lambda)$= relative humidity (%)

2. Coordinate Conversion (m/s → degrees/hour)

Conversion factors (approximate, constant):

- 1° latitude ≈ 111 km → $\frac{d\phi}{dt} = \frac{v \times 3600}{111000}$°/hour
- 1° longitude ≈ 111 km × cos(ϕ) → $\frac{d\lambda}{dt} = \frac{u \times 3600}{111000 \times \cos(\phi)}$°/hour

In code:

$$\frac{d\phi}{dt} = \frac{v}{111.0}, \frac{d\lambda}{dt} = \frac{u}{111.0 \times \max(\cos(\phi), 10^{-6})}$$

3. Hazard-Based Hourly Survival Probability

Survival is multiplicative over time. Hourly survival probability $s(t)$:

Hard biological cutoffs (Culex-specific):

- If $T_C < 5$°C → $s = \epsilon$(near-zero, inactivity)
- If $T_C > 40$°C → $s = \epsilon$(lethal heat)

where $T_C = T - 273.15$(°C), $\epsilon = 10^{-6}$.

Baseline hourly mortality hazard $m = 0.01$.

Penalties (quadratic outside optima):

- Cold: if $T_C < 15$°C → $m{+}= 0.08 \left(\frac{15-T_C}{10}\right)^2$
- Heat: if $T_C > 30$°C → $m{+}= 0.05 \left(\frac{T_C-30}{10}\right)^2$
- Low RH: if RH < 30% → $m{+}= 0.3 + 0.02 \left(\frac{30-RH}{30}\right)^2$
- Mid RH: if 30% ≤ RH < 70% → $m{+}= 0.02 \left(\frac{70-RH}{40}\right)^2$
- High RH: if RH > 90% → $m{+}= 0.15$
- Wind stress: if $V_h = \sqrt{u^2 + v^2} > 10$m/s → $m{+}= 0.02 \left(\frac{V_h-10}{10}\right)^2$

Final hourly survival:

$$s = \max(\epsilon, \exp(-m))$$

Cumulative survival up to time t: $S(t) = \prod_{k=0}^{t} s(k)$

4. 4th-Order Runge-Kutta (RK4) Integration

Trajectory integrated with $\Delta t = 1$ hour, max 240 hours.

Velocity function:

$$\mathbf{F}(t, \mathbf{X}) = \left[\frac{d\phi}{dt}, \frac{d\lambda}{dt}\right]$$

RK4 steps (standard):

$$\mathbf{k}_1 = \mathbf{F}(t_n, \mathbf{X}_n)$$

$$\mathbf{k}_2 = \mathbf{F}(t_n + 0.5, \mathbf{X}_n + 0.5\mathbf{k}_1)$$

$$\mathbf{k}_3 = \mathbf{F}(t_n + 0.5, \mathbf{X}_n + 0.5\mathbf{k}_2)$$

$$\mathbf{k}_4 = \mathbf{F}(t_n + 1, \mathbf{X}_n + \mathbf{k}_3)$$

Update:

$$\mathbf{X}_{n+1} = \mathbf{X}_n + \frac{1}{6}(\mathbf{k}_1 + 2\mathbf{k}_2 + 2\mathbf{k}_3 + \mathbf{k}_4)$$

Longitude wrapped: $\lambda = (\lambda + 180)\mathrm{mod}\ 360 - 180$.

At each step, survival updated: $S_{n+1} = S_n \times s_{n+1}$.

5. Landing and Korea Positions Collection

South Korea domain: strict land polygon (from sig.shp, unary union).

Trajectory "landed" if:

- Enters polygon at any step
- AND at a step inside polygon, interpolated $w(t+1) > 0$(downward motion)

All positions inside Korea (after entry) are collected with weight = current cumulative survival $S(t)$.

If never meets landing condition → discard trajectory.

6. Survival-Weighted Dispersal Risk on Grid

~1 km grid over Korea (resolution 0.009°).

Dispersal risk grid:

$$D_{i,j} = \sum_{\text{landed trajectories}} \sum_{\text{positions in cell (i,j)}} S(t)$$

Normalized: $D \leftarrow D/D_{\max}$(if >0).

Smoothed:

$$D_{\text{smooth}} = \text{GaussianFilter}(D, \sigma \approx 3 \text{ pixels} \approx 3 \text{ km})$$

Re-normalized and masked to land polygon only.

7. Exposure Risk

Interpolated grids (nearest neighbor):

- $L(\phi, \lambda)$: interpolated LSDV suitability [0-1]
- $H(\phi, \lambda)$: normalized livestock density (cattle + buffalo) [0-1]

Final exposure risk:

$$E(\phi, \lambda) = D_{\text{smooth}} \times L \times H$$

Normalized: $E \leftarrow E/E_{\max}$(if >0), masked to land.

8. Risk Classification

On non-zero cells of E (or D_smooth for pathway risk):

- Percentiles of positive values: q50, q75, q90
- Class:

- 1: $0 < E \leq q50$ (Low)
- 2: $q50 < E \leq q75$ (Moderate)
- 3: $q75 < E \leq q90$ (High)
- 4: $E > q90$ (Very High)

9. Origin Sampling

Origins weighted by combined suitability $S_{\text{origin}} = S_{\text{vector}} \times S_{\text{LSDV}}$(normalized, threshold >0 enforced via masking).

Balanced sampling: 1200 origins per source country (China, Japan, Russia FE, North Korea).

Summary Table of Key Parameters

**Symbol**	**Meaning**	**Value/Range**
Δt	RK4 time step	0.5 hour
T_max	Maximum duration	240 hours
ε	Minimum survival	1e-6
σ_smooth	Gaussian smoothing	~3 km
GRID_RES	Korea grid resolution	0.009° (~1 km)
S_vector	Vector (Culex) suitability	[0-1]
S_LSDV	LSDV environmental suitability	[0-1]
H	Livestock density (normalized)	[0-1]
